# Atomic scale analysis of Zn^2+^ storage in robust tunnel frameworks†

**DOI:** 10.1039/d3sc03380e

**Published:** 2023-07-27

**Authors:** Kaiyue Zhu, Hongxin Wang, Weikang Jiang, Weili Xie, Xu Li, Zhenghao Jia, Weishen Yang

**Affiliations:** a State Key Laboratory of Catalysis, Dalian Institute of Chemical Physics, Chinese Academy of Sciences Dalian 116023 China yangws@dicp.ac.cn; b University of Chinese Academy of Sciences Beijing 100049 China; c Department of Chemical Physics, University of Science and Technology of China Anhui 230026 Hefei China; d Division of Energy Research Resources, Dalian Institute of Chemical Physics, Chinese Academy of Sciences 457 Zhongshan Road Dalian 116023 China

## Abstract

Realizing rapid and reversible Zn^2+^ storage at the cathode is imperative for the advancement of aqueous Zn-ion batteries (ZIBs), which offer an excellent option for large-scale electrochemical energy storage. However, owing to limitations of the structural stability of previously investigated frameworks, the Zn^2+^ storage processes remain unclear, thus hindering progress towards the above goal. Herein, we present the novel application of MoVTe oxide with an M1 phase (MVT-M1) as a potential cathode material for ZIBs. MVT-M1 features broad and robust tunnels that facilitate reversible Zn^2+^ insertion/extraction during cycling, as well as rich redox centers (Mo, V, and Te) to aid in charge redistribution, resulting in good performances in ZIBs. The exceptional resilience of MVT-M1 to high-energy electron beams allows for direct observation of Zn^2+^ insertion/extraction at the atomic scale within the tunnels for the first time using high-angle annular dark field scanning transmission electron microscopy; the storage location of zinc ions within the cathode is accurately determined layer by layer from the surface to the bulk phase by employing time-of-flight secondary ion mass spectrometry. Additionally, solvent molecules (H_2_O and methanol) are also found inside the tunnels along with Zn^2+^. Due to the broader heptagonal tunnels and Te ions in the hexagonal tunnels, MVT-M1 exhibits good cycling stability, outperforming MoVTe oxide with the M2 phase (no heptagonal tunnels) and MoV oxide with the M1 phase (no Te). These findings hold significant importance in advancing our understanding of the Zn^2+^ storage mechanism and enable the design of novel materials specifically optimized for efficient Zn^2+^ storage.

## Abstract 2

A rapid and reversible Zn^2+^ insertion/extraction process is achieved in MoVTe oxide with the M1 phase owing to the broad tunnels and robust structure. Moreover, Zn^2+^ insertion along with the solvent water into the tunnels is demonstrated and observed at the atomic scale for the first time.

## Graphical abstract

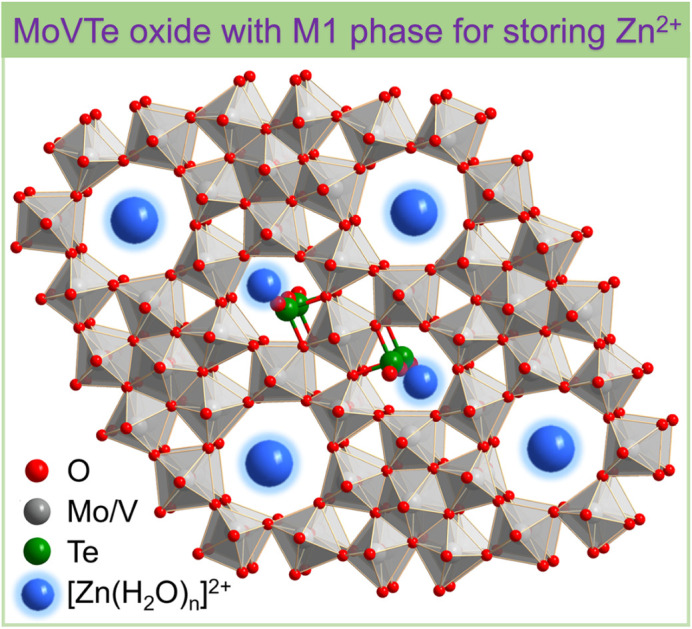

## Introduction

The deployment of renewable energy systems in the current fossil-fuel-dominated energy infrastructure requires the use of large-scale energy storage (LSES) technologies.^1^ Rechargeable batteries present an intriguing and significant option for LSES.^2^ However, although the rechargeable battery market is currently dominated by lithium-ion batteries (LIBs), the use of LIBs in LSES is still under constant debate because of the availability of lithium and a potential increase in its cost, together with the safety concerns and environmental challenges arising from volatile, flammable, and toxic organic electrolytes.^3^ Therefore, alternative batteries employing abundant elements and safe electrolytes are highly desirable.

Among these alternatives, aqueous Zn batteries stand out as ideal alternatives owing to the high abundance and low cost of Zn, together with safe electrolytes.^4^ In addition, the high ionic conductivity of the aqueous Zn^2+^ electrolyte enables fast charging and high power densities, which are crucial features for LSES.^2*a*,5^ More importantly, the redox potential of Zn/Zn^2+^ is within the electrochemical stability window of water, enabling Zn metal to be used directly as an anode in aqueous electrolytes. Earlier applications of alkaline Zn batteries, such as Zn–MnO_2_, Zn–Ni, Zn–Ag_2_O, and Zn–air batteries, suffered from issues such as dendrite formation, corrosion, and irreversible by-products at the Zn anode, which ultimately limited their lifespans.^6^ Nevertheless, these issues can be alleviated by using near-neutral electrolytes, thus guaranteeing a longer lifespan. Therefore, aqueous Zn-ion batteries with near-neutral electrolytes have gained significant attention for use in LSES.

Despite the unique superiority of the anode and electrolyte in Zn-ion batteries (ZIBs), identifying a high-performance host for Zn^2+^ is difficult owing to the substantial coulombic interaction between the incoming Zn ions and host lattices within the cathode.^7^ Numerous studies on ZIB cathodes have been conducted on primary manganese- and vanadium-based materials with layered or tunnel structures, along with minor Prussian-blue analogues, MoS_2_, and organic compounds.^8^ Therefore, significant progress has been made in cathode technology to improve the performance of rechargeable aqueous ZIBs; however, their structural stability must be examined further for the commercialisation of ZIBs. For example, Prussian blue analogues, MoS_2_, and organic compounds exhibit limited capacities and poor cycling stabilities.^8*d*,9^ In contrast, layered materials such as hydrated vanadium oxides (V_2_O_5_·*n*H_2_O and Na_2_V_6_O_16_·*n*H_2_O) and tunnel materials such as VO_2_ and α/β-MnO_2_ deliver high capacity but undergo phase transformation or dissolution during intercalation/de-intercalation of the Zn ions.^10,11^ Specifically, MnO_2_ with a tunnel structure suffers from severe dissolution when cycled in aqueous ZIBs.^11*c*^ VO_2_ and V_2_O_3_ also undergo phase transformation to layered V_2_O_5_·*n*H_2_O during charging.^11*b*,*e*^ While layered M_*x*_V_2_O_5_·*n*H_2_O and M_*x*_V_6_O_16_·*n*H_2_O (M: Zn, Na, K, *etc.*) exhibit higher capacity and better stability, the interlayer water is prone to loss under the bombardment of ultrahigh energy electron beams. Therefore, the structural instability of previously reported cathode materials hinders the observation of the critical Zn^2+^ storage process, which is crucial for guiding the design of high-performance hosts for Zn^2+^. In addition to robust structures, a good cathode material must also exhibit rapid kinetics of Zn^2+^ diffusion in the host. As a cation is inserted into the host lattice, a neighbouring transition metal ion in the host framework must be reduced to maintain the overall charge neutrality. Sufficient redox centres are necessary to enable fast charge neutrality, thereby lowering the diffusion barrier of Zn^2+^. Therefore, novel cathode materials possessing robust structures and a strong charge redistribution ability are urgently required to overcome the structural degradation in host lattices and slow diffusion kinetics of Zn^2+^.

Based on the criteria mentioned above, a robust tunnel framework of MoVTe oxide with an M1 phase (MVT-M1), mainly composed of Mo/V–O octahedra, was studied for the first time as a ZIB cathode to host Zn^2+^ ions. The highly stable Mo/V–O octahedra are corner-linked to form robust hexagonal and heptagonal channels with diameters of ∼5 and 6 Å, providing facile pathways for Zn^2+^ migration with minimum steric hindrance and coulombic interactions. In addition to the redox processes from Mo and V, finite (TeO_4_) chains occupying the hexagonal channels could be reduced with the insertion of Zn^2+^, thus enabling the accessibility of Zn^2+^ into the hexagonal channels and facilitating Zn^2+^-migration kinetics. Atomic scale analysis was conducted on the tunnel structures of MVT-M1 at various states of charge to investigate the Zn^2+^ insertion sites. The effects of Mo, V, Te, the solvent, and the tunnel shape on the performance were determined to demonstrate the critical parameters affecting the performance of ZIBs, thus providing valuable insights into the optimization and design of highly efficient materials for Zn^2+^ storage.

## Results and discussion

### Synthesis and characterization of MoVTe oxide with an M1 phase

**Fig. 1:**
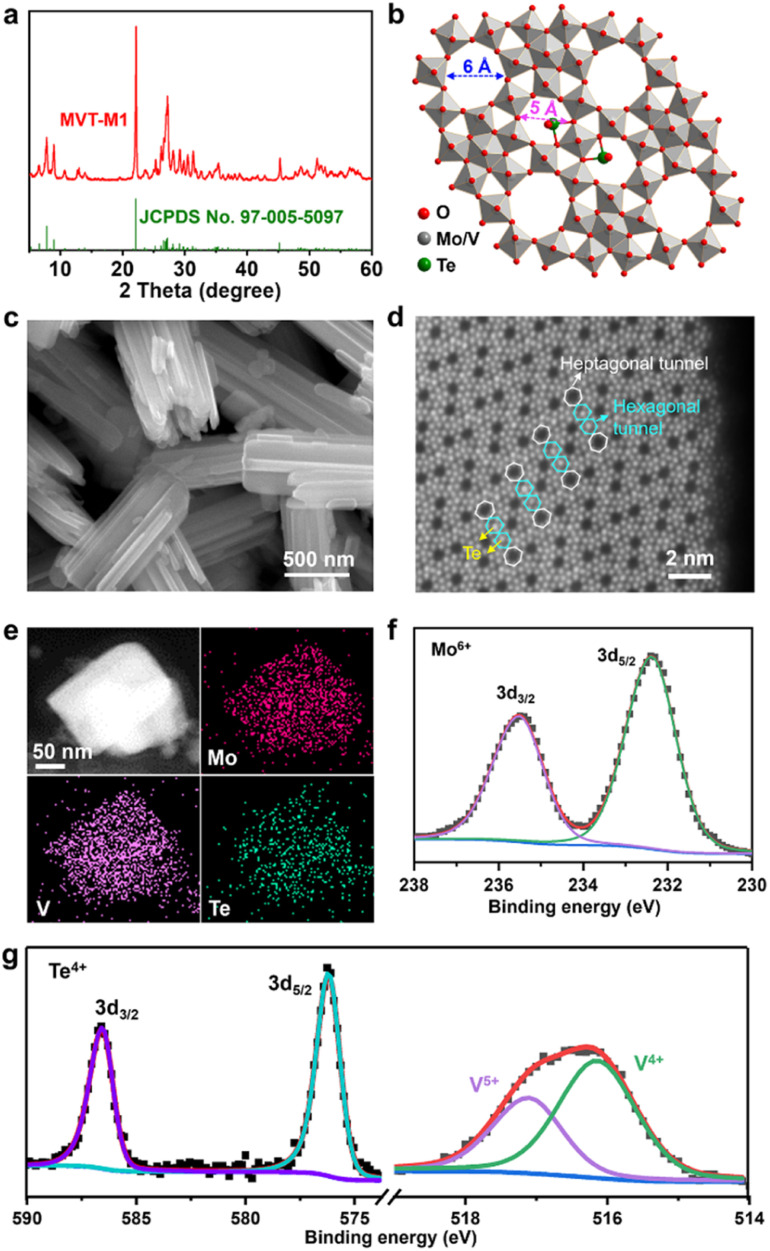
Structural, morphological, and compositional characterizations of MVT-M1. (a) Experimental and standard XRD patterns; (b) crystal structure; (c) SEM image; (d) HAADF-STEM image; (e) STEM image and elemental mapping; and (f) and (g) XPS spectra for the as-prepared MVT-M1. JCPDS no. 97-005-5097 is the standard card of (TeO)_0.94_(Mo_7.82_V_1.18_Nb)O_28_ with the M1 phase.

The MoVTe oxide with an M1 phase (denoted as MVT-M1) was synthesised using the hydrothermal method developed in a previous study (see details in the Methods section).^12^ In Fig. 1a, the X-ray diffraction (XRD) peaks for the as-prepared sample are well indexed to (TeO)_0.94_(Mo_7.82_V_1.18_Nb)O_28_ with the M1 phase (space group: *Pba*2; JCPDS No. 97-005-5097), confirming the formation of the targeted orthorhombic MVT-M1. The crystal structure of MVT-M1 (Fig. 1b) contains hexagonal and heptagonal tunnels with diameters of ∼5 and 6 Å, respectively.^13^ The two types of tunnels are large enough to accommodate Zn^2+^ ions and water molecules. Notably, Te is present in the hexagonal tunnels. Scanning electron microscopy (SEM) images show a prismatic morphology consisting of several coalescent rod-like crystallites (Fig. 1c and S1†). The primary crystallites are nanorods with a width of ∼100 nm and a length of ∼2 μm, while the small single crystallites grow with obvious grain boundaries into large polycrystalline structures with a width of ∼500 nm and a length of ∼2 μm. The nitrogen adsorption/desorption isotherms and pore-size distribution in Fig. S2† show that the Brunauer–Emmett–Teller (BET) surface area of the MVT-M1 sample is 23 m^2^ g^−1^ with large pores of around 13 nm arising from the interparticle space. Typical high-angle annular dark field (HAADF) imaging in scanning-transmission-electron-microscopy (STEM) mode was used to characterize the synthesized M1 phase at the atomic level (Fig. 1d and S3†). The HAADF image shows ordered hexagonal and heptagonal tunnels, and Te is present in the hexagonal tunnels, corresponding well with the crystal structure. Energy-dispersive spectroscopy (EDS) mapping demonstrates a uniform distribution of Mo, V, Te, and O in the rods (Fig. 1e and S4†). X-ray photoelectron spectra (XPS) confirm the oxidation states of the metal oxides; the oxidation states of Mo and Te are +6 and +4, respectively, whereas V exists in the oxidation states of +4 and +5 (Fig. 1f and g). The molecular formula of MVT-M1 was estimated to be MoV_0.41_Te_0.12_O_4_ based on the results of X-ray fluorescence (XRF) spectroscopy (Table S1†).

### The Zn^2+^ storage capability of MVT-M1

**Fig. 2:**
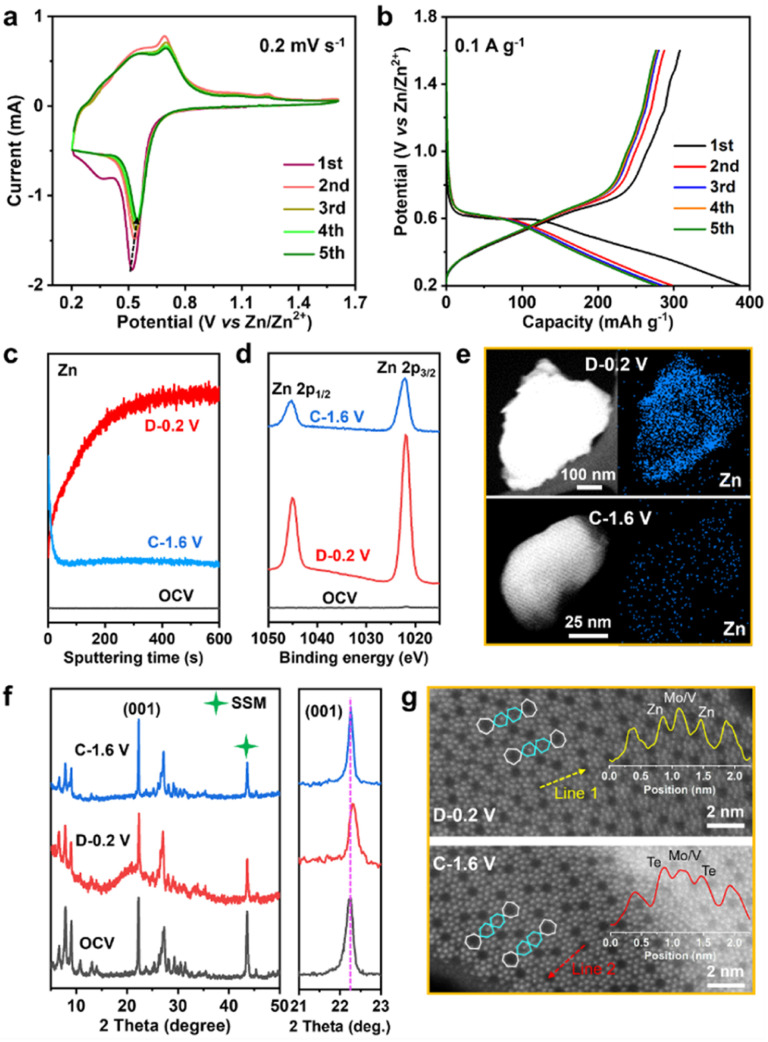
Compositional and structural analysis of the MVT-M1 cathode in various states. (a) CV curves at a scan rate of 0.2 mV s^−1^ and (b) galvanostatic discharge/charge curves during the first five cycles at 0.1 A g^−1^ for ZIBs with MVT-M1 cathodes. (c) Depth profiles of Zn ions obtained by sputtering for the MVT-M1 cathodes at various SOCs. The depth of the sputtering for 600 s is approximately 10 μm. (d) Zn-2p XPS spectra. (e) STEM image and Zn mapping, (f) *ex situ* XRD patterns, and (g) HAADF-STEM images for the MVT-M1 cathode in various states. The insets in (f) show the corresponding atomic intensities along lines 1 and 2.

The Zn^2+^ storage ability of MVT-M1 as a cathode was evaluated in coin-cell-type ZIBs consisting of a Zn metal anode and an aqueous 2 M Zn(OTf)_2_ electrolyte. Cyclic voltammetry (CV) curves show a sharp reduction peak at 0.52 V for Zn^2+^ insertion and a wide oxidation peak ranging from 0.54 to 0.7 V for Zn^2+^ extraction (Fig. 2a). Compared to the 1st CV curve, the Zn^2+^ insertion process becomes more facile in the subsequent 2nd–5th cycles owing to an increase in the Zn^2+^ insertion potential from 0.52 to 0.55 V. Other than a slight decrease in the reduction current, the nearly identical overlap of the subsequent 2nd–5th cycles indicates good reversible properties of Zn^2+^ insertion/extraction into/from the MVT-M1 tunnel structures. The single and sharp reduction peak suggests rapid charge redistribution during Zn^2+^ insertion into MVT-M1. The galvanostatic discharge/charge profiles of the first five cycles at 0.1 A g^−1^ are shown in Fig. 2b. In the first cycle, the discharge capacity is higher than the charge capacity indicating that the inserted ions could not be fully extracted during charging, while a similar capacity is observed during charging and discharging in the subsequent four cycles, suggesting a reversible ion insertion/extraction process after the activation of the first cycle. It is worth mentioning that the MVT-M1 structure has achieved a notable capacity of approximately 380 mA h g^−1^, which is slightly lower than the theoretical capacity (450 mA h g^−1^) expected based on the redox of Mo^6+^/Mo^4+^, V^5+^/V^3+^, Te^4+^/Te^0^. This observation suggests that further optimization can be implemented to enhance the capacity of MVT-M1. To elucidate the insertion/extraction of Zn ions in MVT-M1, XPS and time-of-flight secondary ion mass spectrometry (TOF-SIMS) were used to analyse the elemental composition of the MVT-M1 cathode at various states of charge (SOCs) (see Fig. 2c, d, S5 and S6†): open-circuit voltage (OCV), full discharged state (*D* −0.2 V), and full charged state (*C*−1.6 V). Compared to the OCV, a significant enhancement in the intensity of the Zn peak in both the bulk (Fig. 2c) and surface (Fig. 2d) of MVT-M1 in the discharged state indicates the insertion of Zn. In contrast, the intensity of the Zn peaks in MVT-M1 in the fully charged state is higher than that in the OCV state but much lower than that in the discharged state, demonstrating that minor inserted Zn^2+^ ions are not released. Similar changes in Zn content in the MVT-M1 particles during cycling are also evidenced by EDS mapping (Fig. 2e and S7–S9†). The reversible appearance/disappearance of Zn(OH)_2_ with some (CF_3_SO_3_)^−^ ions in the interlayers (abbreviated as Zn(OH)_2_–S) on the surface of the MVT-M1 cathode at the discharged state and charged state is an indication of H^+^ insertion/extraction into/from MVT-M1 (Fig. S10 and S11†), as the decrease in H^+^ concentration in the electrolyte results in an increase in OH^−^ concentration and ultimately the formation of Zn(OH)_2_–S at the electrode interface.^10*c*,14^

It should be noted that Mo, V, and Te signals could not be detected by XPS in the fully discharged MVT-M1 electrode (Fig. S6†) due to the interference of Zn(OH)_2_–S nanoflakes on the electrode surface (Fig. S10b†). This is because XPS is only capable of detecting a depth of ∼5 nm. To identify the valence states of Mo, V, and Te in the fully discharged MVT-M1 electrode, we used ion etching to remove the nanoflakes. The XPS spectra of the MVT-M1 cathode in the discharged state after ion etching (*D*−0.2 V after ion etching) clearly demonstrated that the valence states of Mo, V and Te were decreased significantly, transitioning from Mo^6+^, V^5+^, V^4+^ and Te^4+^ to Mo^4+^, Mo^5+^, V^4+^, V^3+^ and Te^0^ (Fig. S12 and S13†).

Given the use of an aqueous 2 M Zn(OTf)_2_ electrolyte with a high ionic conductivity (∼60 mS cm^−1^) and a low current density (0.1 A g^−1^, Fig. 2b), it is reasonable to assume that the effects of electrolytes and reaction kinetics are minimal. Hence, the low potential plateau observed in MVT-M1 can be attributed to the inherent redox properties of the material. According to the standard electrode potential meter, the potential plateau observed in the Zn||MVT-M1 battery is determined by the difference in redox potential between MVT-M1 and Zn. CV curves (Fig. 2a) and XPS spectra (Fig. S13†) reveal that Mo, V, and Te undergo reduction during discharge, with only one reduction peak appearing in the CV. This suggests that the charge distribution within the tunnel frameworks is rapid, facilitating the coreduction of Mo, V, and Te.

To identify changes in the crystal structure and tunnels of the MVT-M1 particles, *ex situ* XRD and HAADF-STEM analyses were performed on MVT-M1 at various SOCs. A comparison of the XRD patterns shows that the crystal structure of MVT-M1 is well maintained, and there is a slight lattice shrinkage when Zn^2+^ is inserted (Fig. 2f). In addition, the HAADF-STEM analysis detected changes in the hexagonal tunnels. Compared with MVT-M1 in the OCV state (Fig. 1d and S14†), the brightness of the atoms in the hexagonal tunnels is significantly lower in the discharged state, which then returns to its original brightness in the charged state, giving a hint of Zn^2+^ insertion into the hexagonal tunnels (Fig. 2g, S15 and S16†). Notably, due to the strong influence of atomic mass on brightness in HAADF-STEM, the lower atomic mass of Zn results in lower brightness compared to Te. So far, it has been difficult to draw a conclusion whether the decreased brightness is resulted from the content decrease in Te ions, the insertion of Zn^2+^ in the hexagonal channels or both of them.^13^ Based on the analysis conducted on Fig. S7 and S8c,† it is evident that Te element is present within the MVT particles after charging, while only a small amount of Zn element is observed. Therefore, it can be inferred that the atoms occupying the hexagonal channels of MVT after charging are primarily composed of Te.

### Identification of the Zn^2+^ insertion sites in MVT-M1

**Fig. 3:**
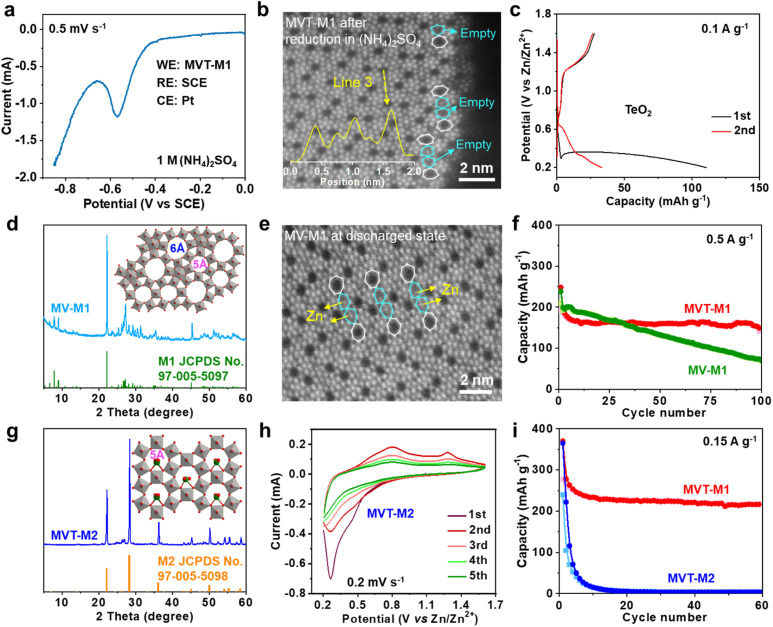
Roles of the Te ions and heptagonal tunnels in MVT-M1. (a) LSV curve at a scan rate of 0.5 mV s^−1^ in 1 M (NH_4_)_2_SO_4_ solution for the MVT-M1 electrode in a three-electrode system with SCE as the RE and Pt wires as the CE. (b) HAADF-STEM image of MVT-M1 after reduction in (NH_4_)_2_SO_4_ solution. (c) Galvanostatic discharge/charge curves for the first two cycles at 0.1 A g^−1^ for ZIBs with TeO_2_ cathodes. (d) Experimental and standard XRD patterns of MV-M1 (inset is the crystal structure of MV-M1). (e) HAADF-STEM image of MV-M1 in the discharged state. (f) Long-term cycling performance of MVT-M1 and MV-M1 at 0.5 A g^−1^. (g) Experimental and standard XRD patterns of MVT-M2 (inset is the crystal structure of MVT-M2). (h) CV curves at a scan rate of 0.2 mV s^−1^ of MVT-M2 in the ZIB. (i) Long-term cycling performance of MVT-M1 and MVT-M2 at 0.15 A g^−1^.

To eliminate interference from Zn, linear sweep voltammetry (LSV) was performed in 1 M (NH_4_)_2_SO_4_ solution with MVT-M1 as the working electrode (WE), a saturated calomel electrode (SCE) as the reference electrode (RE) and Pt wires as the counter electrode (CE) (Fig. 3a). The LSV curve at a scan rate of 0.5 mV s^−1^ shows an obvious reduction peak at approximately −0.56 V *vs.* SCE. After the reduction of MVT-M1 in the (NH_4_)_2_SO_4_ solution, the brightness of the atoms in hexagonal tunnels is also significantly lower (Fig. S17†) than that in pristine MVT-M1 (Fig. 1d and S14†), with some hexagonal tunnels present near the lateral surface being empty (Fig. 3b), directly suggesting that Te is removed from the hexagonal tunnels during reduction. The reduction of Te^4+^ in the hexagonal tunnel is evidenced by the Zn^2+^ storage capability (110 mA h g^−1^) of TeO_2_ (Fig. 3c). The XRD patterns show that TeO_2_ is reduced to Te metal after discharge (Fig. S18†). To further demonstrate the vital role of Te ions in the hexagonal tunnels, we studied MoV oxides with the M1 phase (denoted as MV-M1), whose crystal structure is similar to that of MVT-M1, except for the absence of Te ions in the hexagonal tunnels (Fig. 3d).^15^ After discharge, bright spots attributed to Zn atoms appear in the hexagonal tunnels of MV-M1 (Fig. 3e). This result clearly demonstrates Zn^2+^ insertion into the hexagonal tunnels. Hence, based on comparative experiments involving the reduction of MVT-M1 in zinc-ion-free electrolytes (such as (NH_4_)_2_SO_4_) and the reduction of MV-M1 (without Te) in Zn^2+^-electrolytes, it can be concluded that the atoms present within the hexagonal channels of discharged MVT-M1 are Zn.

In addition, the long-term cycling stability of MV-M1 is poorer than that of MVT-M1, suggesting that the Te ions in the hexagonal tunnels play a vital role in stabilizing the structures (Fig. 3f). To understand the role of the heptagonal tunnels, MVT oxides with the M2 phase (denoted as MVT-M2) containing only hexagonal tunnels but no heptagonal tunnels were also synthesised to study the Zn^2+^ storage performance (Fig. 3g).^16^ The CV (Fig. 3h) and galvanostatic curves (Fig. 3i and S19†) of MVT-M2 show an irreversible Zn^2+^ insertion/extraction process and a sharply decreased capacity from 365 to 15 mA h g^−1^ (at 0.15 A g^−1^) within 10 cycles. The better cycling stability of MVT-M1 compared with those of MVT-M2 and MV-M1 (Fig. 3i and f) clearly demonstrates the stabilizing role of Te ions and heptagonal tunnels. Unlike the structure of MVT-M2, which is destroyed during cycling (Fig. S19†), the structure of MVT-M1 is still well maintained after being soaked for 12 months or 60 cycles at 0.15 A g^−1^ (Fig. S20†), further demonstrating the excellent structural stability of MVT-M1. Note that the hexagonal and heptagonal tunnels in MVT-M1 and MV-M1 in the discharged state (Fig. 2g and 3b, e) become more irregular than those in MVT-M1 in the OCV and charged states, also indicating the reduction of Mo/V atoms and insertion of Zn^2+^ when discharged. The results also indicate that Zn^2+^ insertion affects the spatial arrangement of the atoms constituting the tunnels. Since no obvious spots are observed in the heptagonal tunnels, the arrangement of the inserted Zn atoms in the heptagonal tunnels may be unordered. Overall, Mo, V, and Te contribute to electron transfer during Zn^2+^ storage, while the heptagonal tunnels and Te ions enable high reversibility of Zn^2+^ storage in MVT-M1.

### The critical role of the solvent water in Zn^2+^ transport

**Fig. 4:**
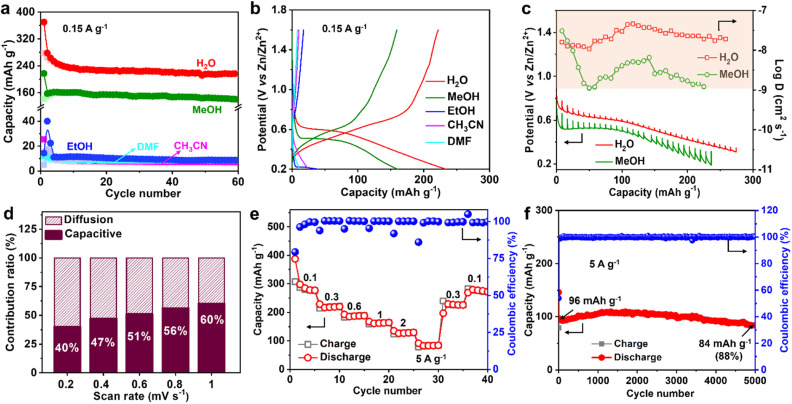
Insertion kinetics and performance of MVT-M1 in ZIBs. (a) Long-term cycling performance and (b) corresponding galvanostatic discharge/charge curves (the 3rd cycle) at 0.15 A g^−1^ for the MVT-M1 cathodes in 0.25 M Zn(OTf)_2_ electrolytes using various solvents. (c) Discharge GITT curves and Zn^2+^ diffusion coefficient of the MVT-M1 cathode at a current density of 50 mA g^−1^ in 1 M Zn(OTf)_2_ electrolyte with water or methanol as the solvent. (d) Capacitive contribution ratio of MVT-M1 at multiple scan rates from 0.2 to 1 mV s^−1^. (e) Rate capability of the MVT-M1 cathode at various current densities. (f) Long-term cycling performance of MVT-M1 at 5 A g^−1^ for 5000 cycles.

The Zn^2+^ ion in the electrolyte is surrounded by water, which produces hydrated zinc ions such as [Zn(H_2_O)_6_]^2+^, therefore, the solvent water may affect Zn^2+^ insertion into MVT-M1.^17^ To study the role of water, the Zn^2+^-storage performance was evaluated in Zn^2+^-based electrolytes (0.25 M Zn(OTf)_2_) using various solvents, such as methanol (MeOH), ethanol (EtOH), acetonitrile (CH_3_CN), and dimethyl formamide (DMF). The discharge capacities at 0.15 A g^−1^ in ethanol, acetonitrile and DMF are 40, 25, and 12 mA h g^−1^, respectively (Fig. 4a, b and S21†), whereas the capacity in methanol is as high as 160 mA h g^−1^, which is only slightly lower than that in water (250 mA h g^−1^). Furthermore, the CVs in different solvents (Fig. S22a†) reveal that the redox potential is greatly influenced by the solvents. The ohmic resistances obtained from electrochemical impedance spectra (EIS) for the coin-type cells in electrolytes using different solvents range from 1.9 to 6 ohms (see Fig. S22b–d†), eliminating the effects from the electrolytes. By showing significantly lower electrolyte resistances from ionic conductivities (Table S2†) than ohmic resistances from EIS (Fig. S22b–d†), it is further confirmed that the discrepancies in capacity are not caused by small differences in ionic conductivities. This is probably because water (kinetic diameter: 3.2 Å) and methanol (4.3 Å) molecules can easily enter the tunnels along with Zn^2+^, while larger molecules such as ethanol (5.2 Å), acetonitrile (6.5 Å) and DMF (5.7 Å) cannot fit through the tunnels. Therefore, for effective performance, the solvent (water and methanol) must be inserted together with Zn^2+^ into the tunnels of MVT-M1. In particular, when MVT-M1 is immersed in electrolytes using MeOH as the solvent, it exhibits a significantly higher capacity of 160 mA h g^−1^ compared to using EtOH, which only yields around 10 mA h g^−1^. Both MeOH and EtOH are capable of contributing H^+^ ions. However, the discrepancy in performance can be attributed to the kinetic diameter of the solvents. MeOH, with a smaller diameter of 4.3 Å, is able to enter the hexagonal channels, whereas EtOH, with a larger diameter of 5.2 Å, fails to do so. This inability to access the tunnels is observed similarly with CH_3_CN and DMF, resulting in underutilization of active sites and subsequently leading to a very low capacity. These results also indicate that the energy-storage process (*i.e.* charge transfer) mainly occurs in the tunnels rather than the particle surface.^18^ It should be emphasized that the morphology and crystal structures of MVT-M1 remain well maintained in electrolytes using EtOH, DMF, and CH_3_CN solvents (Fig. S23 and S24†). This underscores the importance of having large interlayer spacing and tunnels in the cathodes of ZIBs. These structural features facilitate efficient ion transport and maximize the utilization of active sites within the bulk material.

Galvanostatic intermittent titration technique (GITT) was also carried out to obtain a deeper insight into the diffusion process.^19^ A series of galvanostatic discharge pulses of 10 min at 50 mA g^−1^, followed by 1 h of relaxation, was performed (Fig. 4c). In the Zn(OTf)_2_ aqueous electrolyte, the Zn^2+^ diffusion coefficient in MVT-M1 using GITT was estimated to be ∼10^−7.5^ cm^2^ s^−1^ (Fig. 4c and S25†), which is much higher than that in the Zn(OTf)_2_ in methanol electrolyte (∼10^−8.2^ cm^2^ s^−1^). Therefore, water acts as a lubricant to transport ions in the tunnels, thereby enabling a high capacity. The observed difference in capacity during the cointercalation of Zn^2+^/MeOH and Zn^2+^/H_2_O is primarily due to two factors. First, the Zn^2+^ ions in the tunnels experience a larger charge-transfer resistance, as illustrated in Fig. S22,† when MeOH solvent is used compared to H_2_O solvent. Second, the diffusion coefficient of Zn^2+^ ions in the tunnels is lower when MeOH is used, as indicated in Fig. 4c. It is important to note that the diffusion coefficient applies to both Zn^2+^ and H^+^ ions collectively, as it is challenging to separate and determine the diffusion efficiency of Zn^2+^ and H^+^ individually. Despite the micrometre-size particles, the Zn^2+^-diffusion coefficient in MVT-M1 is also higher than or at least comparable to other nanometre-sized layered and tunnel cathodes (*e.g.*, V_2_O_5_·*n*H_2_O, 10^−9^ to 10^−12^ cm^2^ s^−1^; Zn_0.25_V_2_O_5_·*n*H_2_O, 10^−9^ to 10^−10^ cm^2^ s^−1^; H_2_V_3_O_8_, 10^−8^ to 10^−10^ cm^2^ s^−1^; Na_2_V_6_O_16_·*n*H_2_O, ∼10^−9^ cm^2^ s^−1^; MoS_2_, 9 × 10^−8^ to 10^−9^ cm^2^ s^−1^; MnO_2_, 10^−12^ cm^2^ s^−1^; VO_2_, 10^−7.5^ cm^2^ s^−1^).^8*g*,10*a*,10*b*,14*a*,20^ This provides solid evidence for the fast insertion of Zn^2+^ and H^+^ into the MVT-M1 tunnels. To understand the intercalation behaviour in MVT-M1, CV measurements were carried out at various scan rates from 0.2 to 1 mV s^−1^ (Fig. S26a†). Fig. S26b† shows *b* values of 0.71 at the reduction peak (peak 1) and 0.82 at the oxidation peak (peak 2), indicating that the storage mechanism in MVT-M1 is a mix of both diffusive and capacitive behaviours.^21^ The capacitive contribution ratios increase from 40% to 60% as the scan rate increases from 0.2 to 1 mV s^−1^ (Fig. 4d and S26†). This confirms that the capacity of MVT-M1 is mainly attributed to charge transfer in the tunnels rather than a redox process on the surface, consistent with the conclusion of the comparative experiments using different solvents.

### Rapid and reversible insertion/extraction of Zn^2+^ in MVT-M1

Benefiting from the rapid diffusion kinetics in the tunnels, MVT-M1 exhibits an excellent rate capability (Fig. 4e and S27†). The discharge capacities in the stable state are 280, 220, 188, 164, 129, and 84 mA h g^−1^ at 0.1, 0.3, 0.6, 1, 2, and 5 A g^−1^, respectively. Specifically, the capacity drops from 280 to 84 mA h g^−1^ (70% reduction) as the current increases from 0.1 to 5 A g^−1^ (5000% increase). In addition, the electrochemical reversibility of MVT-M1 is also illustrated by the rate performance (Fig. 4e), where the capacity is shown to recover fully at 0.3 A g^−1^ and 0.1 A g^−1^ after cycling at a high current of 5 A g^−1^. The Ragone plot (energy density *vs.* power density) of the Zn||MVT-M1 full cell in Fig. S28† shows energy/power densities of 197 W h kg^−1^/75 W kg^−1^ at 0.1 A g^−1^ (based on the active materials), comparable to that of tunnel-structured MnO_2_ and Prussian blue analogues.

It should be noted that there was a slight decrease in capacity in the initial few cycles (Fig. 3f, i and 4e, f), as some inserted ions could not be extracted from the tunnels. After the initial cycles, MVT-M1 exhibits reversible insertion and extraction processes. At a low current of 0.15 A g^−1^, the capacity decreases from 234 to 216 mA h g^−1^, indicating a capacity retention of 92% at the end of 50 cycles (Fig. 3i). At a high current of 5 A g^−1^, the capacity decreases from 96 mA h g^−1^ to 84 mA h g^−1^, indicating a capacity retention of 88% after 5000 cycles (Fig. 4f). Table S3† shows that the performance of MVT-M1 is comparable to some representative cathode materials utilized in ZIBs. MVT-M1 displays excellent Zn^2+^/H^+^ insertion/extraction reversibility and structural stability at both low and high rates. Overall, the tunnel MVT-M1 oxides possess unique advantages in terms of broad tunnel size and robust structure, thus enabling rapid diffusion kinetics and high reversibility.

Based on the SEM images depicted in Fig. S29,† it can be observed that the overall morphology of the MVT-M1 cathode remains unchanged. However, the electrode surface shows the formation of nanoflakes consisting of Zn(OH)_2_–S. The presence of these nanoflakes suggests that the inserted ions are not completely extracted during the charging process, which is consistent with the observed decrease in performance during cycling. As shown in Fig. S20c and S30a,† there are no Te or TeO_2_ peaks visible in the XRD after cycling, indicating that Te may remain within the structure. As displayed in Fig. 2a and S18b,† the reduction potentials of Te^4+^ in MVT-M1 and TeO_2_ are notably distinct in CV, but the CV curves after cycling (Fig. S30b†) are similar to those at the initial cycles (Fig. 2a), further indicating that Te remains in the structures during cycling. Furthermore, the cycling performance of MVT-M1 is more stable than that of MV-M1 (which lacks Te in the structures), indicating that the Te within the tunnel structures plays a crucial role in maintaining the structures. In addition, Fig. S31† shows that the coin-type Li||MVT-M1 battery in 1 M LiPF_6_ in ethylene carbonate and diethyl carbonate (50/50 v/v) delivers a high capacity of 480 mA h g^−1^ at a current density of 0.1 A g^−1^, indicating the potential of MVT-M1 in other batteries.

Despite the current performance of MVT-M1 falling short of practical application requirements, it possesses specific advantages that make it a noteworthy choice. Firstly, in comparison to hydrated layered V-based cathode materials, the tunnel structure of MVT-M1 is better suited for observing the insertion and extraction of zinc ions during the discharge and charge processes. This is because the interlayer water present in layered V-based materials tends to be lost under the intense energy of electron beams, making it difficult to study the ion dynamics.^11*a*,22^ Secondly, other tunnel structure materials such as MnO_2_, VO_2_, and V_2_O_3_ face challenges when cycled in aqueous ZIBs. MnO_2_ experiences significant dissolution, while VO_2_ and V_2_O_3_ undergo phase transformations to layered V_2_O_5_·*n*H_2_O during charging.^11*b*,11*c*,11*e*,23^ In comparison, MVT-M1 emerges as the most suitable material for investigating the atomic-scale insertion and extraction of Zn^2+^ during discharge and charge processes using HAADF-STEM. This aspect is crucial for comprehending the Zn^2+^ storage mechanism and designing novel materials tailored for efficient Zn^2+^ storage.

## Conclusions

In summary, orthorhombic MVT-M1 with a broad and robust tunnel structure delivers excellent Zn^2+^ storage performance. Mo, V, and Te are involved in charge redistribution during the insertion of Zn^2+^ ions, which favours charge transfer. In addition, the broad tunnels (5–6 Å) and water co-insertion (acting as lubrication) are favourable for ionic diffusion, thus enabling high rate capability. Benefiting from the robust structure and ultra-large heptagonal tunnels, stable cycling performance can be obtained in ZIBs using an MVT-M1 cathode. Combined with STEM mapping and TOF-SIMS, HAADF-STEM images demonstrate that Zn^2+^ is inserted into the hexagonal and heptagonal tunnels. TOF-SIMS clearly shows that Zn(OH)_2_–S (which reflects the insertion of H^+^) appears at the interface of the electrode and bulk electrolyte rather than at the macropores in the bulk of the electrode. Overall, the fundamental insights gained in this study deepen the current understanding of the Zn^2+^ storage mechanism and enable the design of Zn^2+^ storage materials and their optimisation. Furthermore, this study demonstrates the excellent Zn^2+^ storage performance of the broad and robust tunnel frameworks of ZIBs, which can be extended to other metal-ion batteries such as Li, Na, K, Mg, and Al.

## Data availability

The data that support the findings of this study are available from the corresponding author upon reasonable request.

## Author contributions

W. Y. and K. Z. conceived and designed this work. K. Z. collected and analyzed data. H. W., W. J., W. X., and X. L. assisted the material synthesis and characterizations. Z. J. performed the HAADF-STEM characterizations. K. Z. and W. Y. wrote the article. W. Y. revised the article and supervised the project. All authors contributed to the discussion of the results.

## Conflicts of interest

There are no conflicts to declare.

## Supplementary Material

SC-014-D3SC03380E-s001
