# Supplementary material for: Atomic scale analysis of Zn2+ storage in robust tunnel frameworks
Source: Chem Sci. 2023 Jul 27;14(33):8889–96. doi: 10.1039/d3sc03380e (PMC10445463; doi:10.1039/d3sc03380e)
Supplement: SC-014-D3SC03380E-s001 [file SC-014-D3SC03380E-s001.pdf]

Electronic Supplementary Material (ESI) for Chemical Science.
This journal is © The Royal Society of Chemistry 2023

# Supporting Information

**Atomic scale analysis of $Zn^{2+}$ storage in robust tunnel frameworks**

Kaiyue Zhu (0000-0002-1446-0337),[ab] Hongxin Wang,[ab] Weikang Jiang,[ac] Weili Xie,[ab] Xu Li,[ab] Zhenghao Jia,[d] Weishen Yang (0000-0001-9615-7421)[*ab]

[a] K. Zhu, H. Wang, W. Xie, X. Li, W. Yang

State Key Laboratory of Catalysis, Dalian Institute of Chemical Physics, Chinese Academy of Sciences, Dalian, 116023, China.

[b] K. Zhu, H. Wang, W. Xie, W. Yang

University of Chinese Academy of Sciences, Beijing, 100049, China.

[c] W. Jiang

Department of Chemical Physics, University of Science and Technology of China, Anhui 230026, Hefei, China

[d] Z. Jia

Division of Energy Research Resources, Dalian Institute of Chemical Physics, Chinese Academy of Sciences, 457 Zhongshan Road, Dalian, 116023, China.

## Experimental Section

*Synthesis of MoVTe oxide with M1 phase (MVT-1)*

MoVTe oxide with the M1 phase is prepared through a hydrothermal method.[1] In a typical procedure, certain amounts of ammonium heptamolybdate hydrate, vanadyl sulfate hydrate and tellurium dioxide with a Mo/V/Te molar ratio of 1/0.5/0.17 were mixed with water under vigorous stirring for 2 h to form a gel in an autoclave with a Teflon liner. Then, the gel was purged with nitrogen for 10 min to replace the air in the autoclave and then held at 175ºC for 72 h. After the reaction, the precipitate was collected and thoroughly washed with deionized water and dried at 80 °C for 12 h. Finally, the dried precipitate was calcinated at 600 °C for 2h with a heating ramp of 5 °C /min to obtain the desired MVT-M1 powders.

*Synthesis of MoV oxide with the M1 phase (MV-M1)*

MV-M1 is synthesized by a procedure similar to MVT-M1 except for no addition of tellurium dioxide and adjustment of the calcination temperature to 500 °C. In addition, the final powders after thermal calcination were washed with $H_2O_2$ (10%) to purify MV-M1.

*Synthesis of MoVTe oxide with the M2 phase (MVT-M2)*

MVT-M2 is synthesized with the help of vacuum rotary evaporation.[1b] In a typical procedure, 2.1 g ammonium heptamolybdate hydrate, 0.75 g ammonium metavanadate ($NH_4VO_3$) and 1.62 g telluric acid ($H_6TeO_6$) with a Mo/V/Te molar ratio of 1/0.5/0.17 were dissolved in water (30 mL) in a rotary evaporation apparatus at 80 °C. After rotating at atmospheric pressure for 2 h, the above orange solution was cooled to 40 °C and then vacuumed to a pressure of ~$10^{-3}$ Pa at 40 °C. Concentrated by rotary evaporation, the dried precursor is obtained. Finally, the desired MVT-M2 powders were obtained by calcinating the red precursor under a $N_2$ atmosphere at 600 °C for 2h with a heating ramp of 3 °C /min.

## Materials Characterization

*Phase and structure determination.*

X-ray diffraction (XRD) patterns of the samples were collected using a Rigaku D/MAX-2500/PC with Cu Kα radiation (λ=1.54 Å at 40 kV and 200 mA). The data were recorded from 5º to 60º with an interval of 0.02º and a scan speed of 5º/min.

*Microstructure.*

The surface morphologies of the samples were captured with the FEI Quanta 200 F. Elemental mapping along with morphology were obtained by a scanning transmission electron microscope (STEM, JEM-ARM200F) equipped with an energy-dispersive X-ray spectrometer (EDS). The atomic tunnels, crystal structures and morphology were acquired by high angle annular dark field (HAADF) imaging in the scanning transmission electron microscopy (STEM) (Titan Themis ETEM G3).

*Chemical Analysis.*

The concentrations of the elements of interest were analyzed by an inductively coupled plasma optical emission spectrometer (PerkinElmer ICP-OES 7300DV) and X-ray fluorescence spectrometer (Zetium).

*Surface chemistry*.

The surface chemical compositions and oxidation states of the elements were analyzed using a Thermofisher Escalab 250 Xi+ spectrometer with Al Ka X-ray radiation (hν=1486.6eV). Prior to this analysis, the cycled electrodes were washed thoroughly with DI water to remove electrolyte residue and then dried in a glove box. All the binding energies were corrected by adventitious C 1s at 284.6 eV.

**Electrochemical Characterization**

*Battery cell assembly*

Electrochemical tests were carried out in CR2032-type coin cells. To prepare a cathode, 60wt% active materials, 26wt% Super-P, and 14wt% polyvinylidene fluoride (PVDF) were thoroughly mixed and dispersed into N-methyl pyrrolidone (NMP). The high ratios of carbon and binder

are just used to ensure insufficient conductivity and adhesive strength. The resultant slurry was then coated uniformly onto a 14 mm diameter stainless steel mesh (SSM), resulting in an ~ 1.2 mg $cm^{-2}$ active mass loading, followed by drying at 100 ºC for 12 h. In a full ZIB cell, zinc foil was used as the anode, 2 M $Zn(OTf)_2$ as the electrolyte, and glass microfiber filters (Whatman, Grade GF/A) as the separator.

*Electrochemical testing*

The CR2032-type coin cells for zinc ion batteries were assembled in air and tested using a LAND battery testing system (CT2001A) within a potential window of 0.2-1.6 V (*vs* $Zn/Zn^{2+}$).

The CR2032-type coin cells for lithium batteries were assembled in Ar-filled glovebox and tested using a LAND battery testing system (CT2001A) within a potential window of 1.0-3.5 V (*vs* $Li/Li^{+}$).

Cyclic voltammetry (CV) measurements were performed in a CR2032-type coin-cell using a VersaSTAT 3F electrochemical workstation. Linear sweep voltammogram (LSV) experiments were performed in a three-electrode configuration using a VersaSTAT 3F electrochemical workstation. A saturated calomel electrode (SCE) and Pt wire were used as reference and counter electrodes, respectively. The galvanostatic intermittent titration technique (GITT) was used to determine ionic diffusivity from a series of galvanostatic discharge pulses of 10 min at 50 mA $g^{-1}$, followed by a 1 h relaxation. The total ionic diffusion coefficient (${D_{ion}}^{GITT}$) is calculated by [2]:

$$ {D_{ion}}^{GITT} = \frac{4L^2}{\pi\tau}\left(\frac{\Delta E_s}{\Delta E_t}\right)^2 \quad (1) $$

where $\tau$ is the constant current pulse time; L corresponds to the ion diffusion length, which equals the thickness of the electrode; $\Delta E_s$ is the change in steady-state voltage during a single-step GITT experiment; and $\Delta E_t$ is the change in cell voltage at a constant current minus IR-loss during each galvanic step.

The ionic conductivities of 0.25 M $Zn(OTf)_2$ electrolytes utilizing different solvents ($H_2O$, MeOH, EtOH, DMF, and $CH_3CN$) were measured by portable conductivity meter (DDBJ-350F).

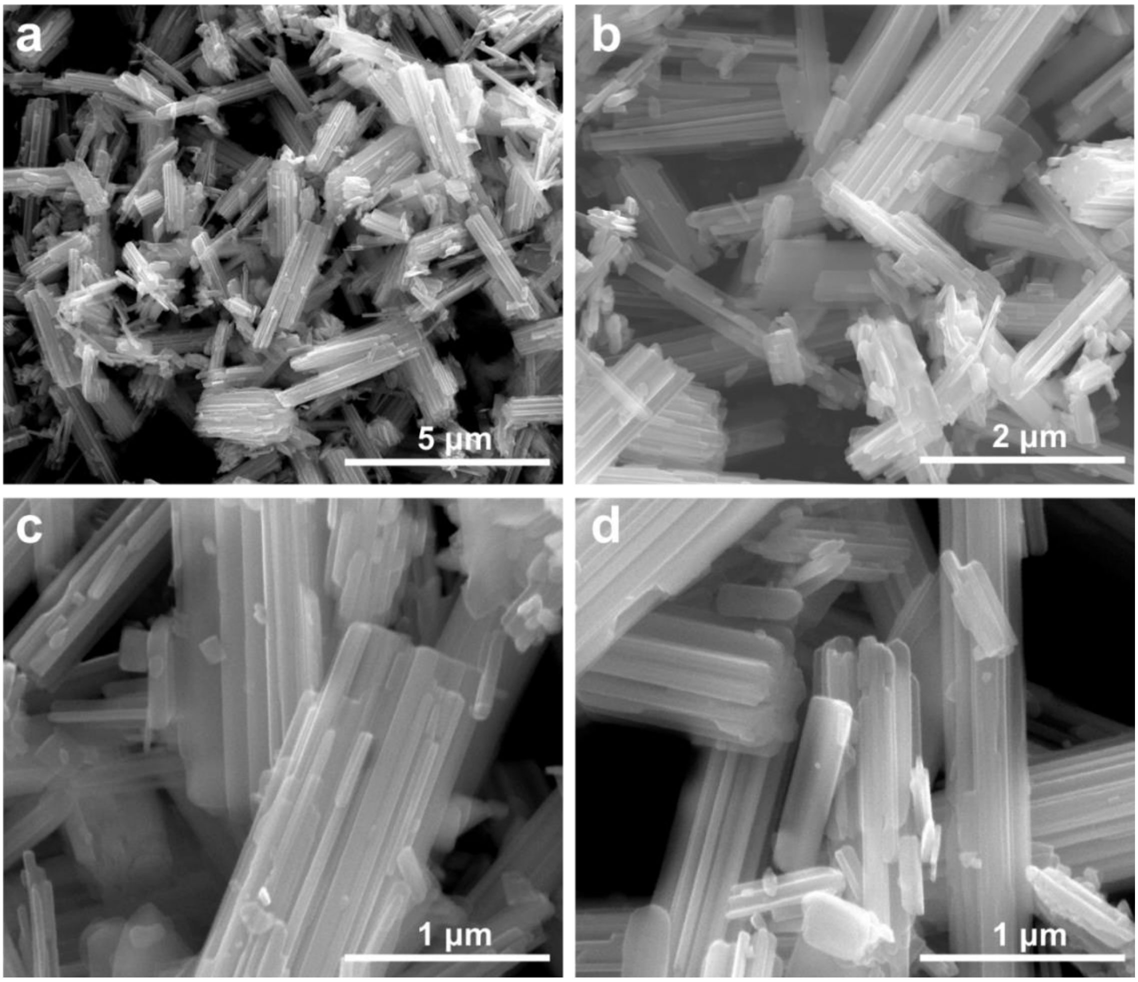


Figure S1. SEM images of as-prepared MVT-M1.

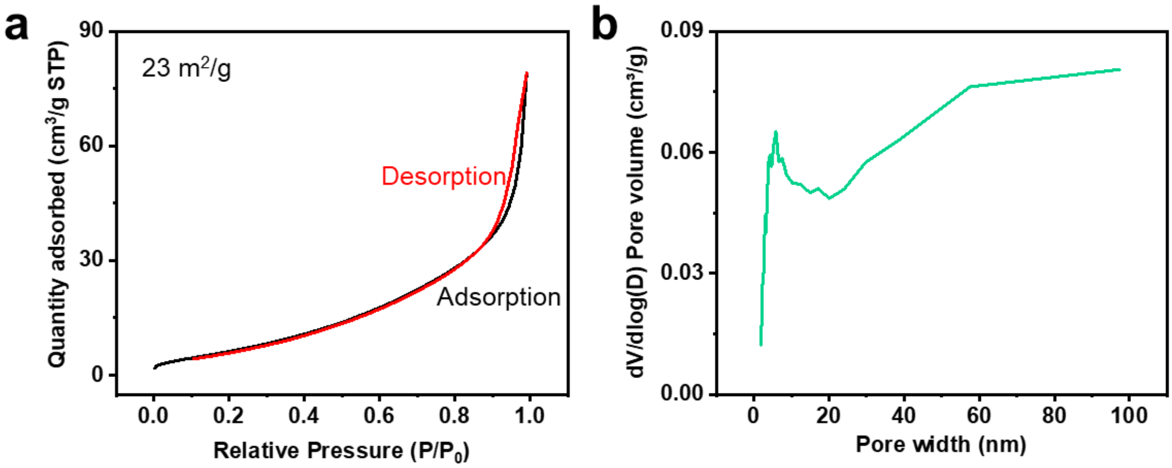


Figure S2. (a) $N_2$ adsorption/desorption isotherms of MVT-M1 powders. (b) The pore size distribution curves of MVT-M1 powders.

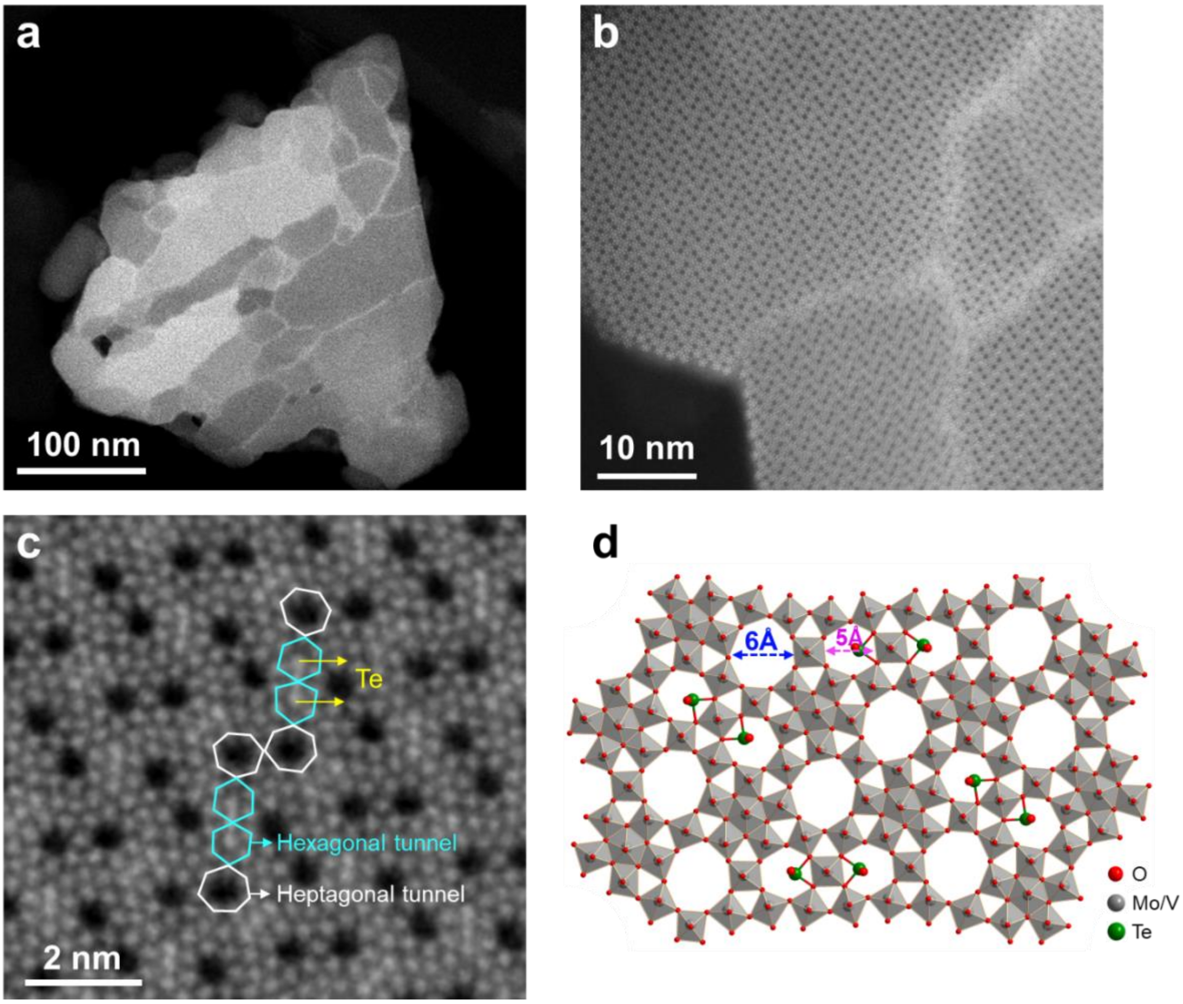


Figure S3. (a) TEM image, (b, c) HAADF-STEM images with different magnifications, (d) the crystal structure of MVT-M1.

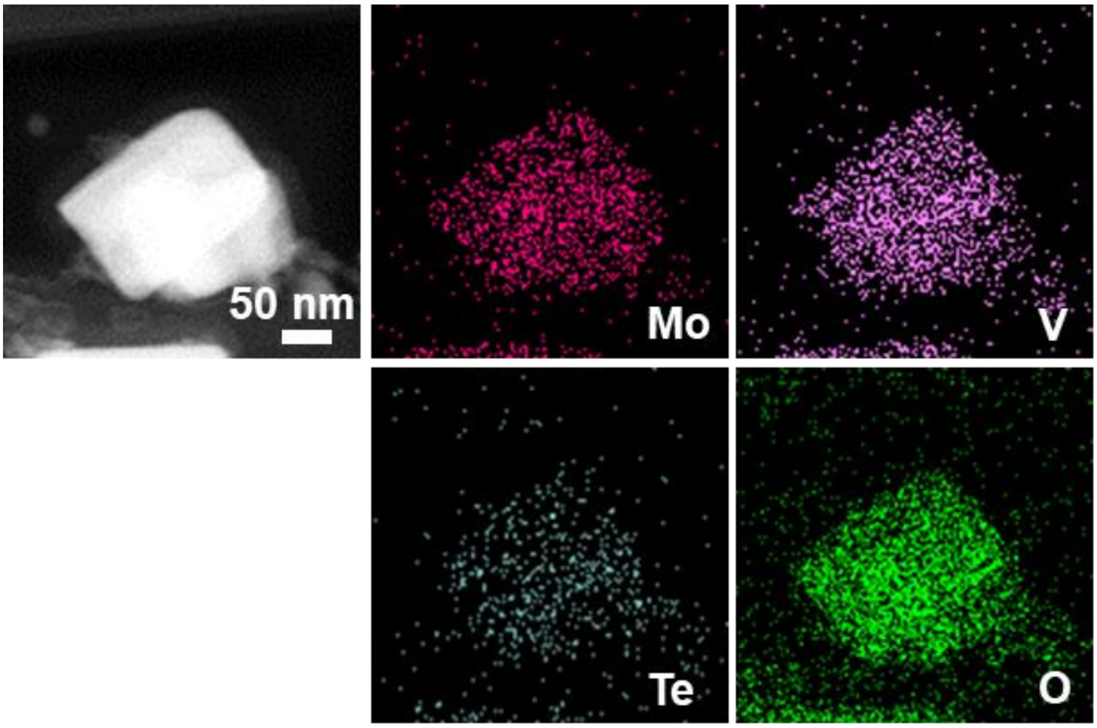


Figure S4. STEM image and corresponding elemental mapping of MVT-M1.

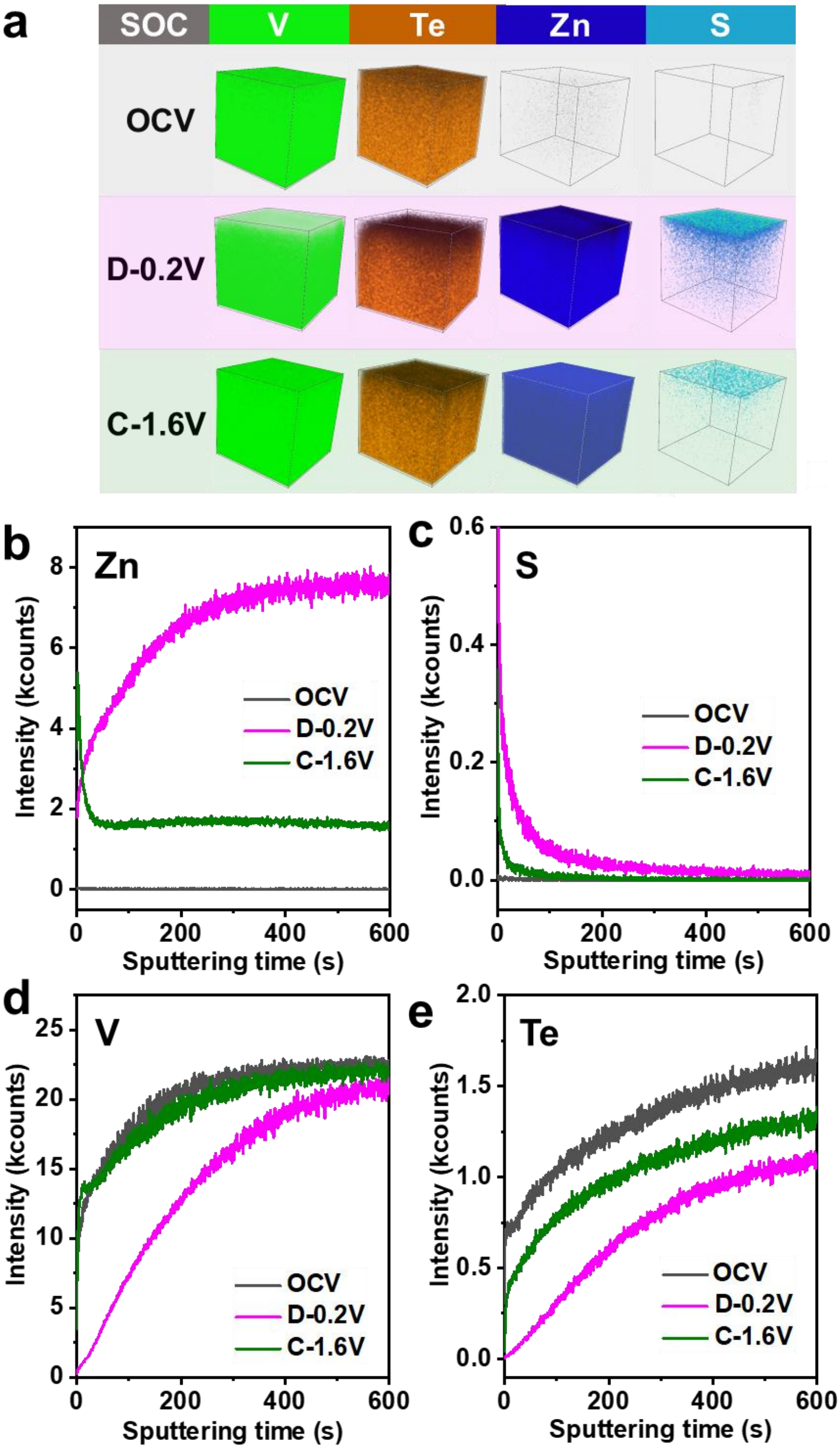


Figure S5. Evolution of the composition distribution with depth for the MVT-M1 cathodes at various SOCs. (a) Three-dimensional (3D) view images of the sputtered volume corresponding to the depth profiles. (b–e) Depth profiles of V, Te, Zn, and S ions obtained by sputtering for the MVT-M1 cathodes at various SOCs. The analysis area is 50 × 50 μm$^2$, and the sputtering depth for 600 s is approximately 10 μm.

To identify the depth distributions of byproducts and Zn inserted into the MVT-M1 cathode, time-of-flight secondary ion mass spectrometry (TOF-SIMS) was performed on the MVT-M1 cathode at various SOCs (Figure S5). For the MVT-M1 cathode in the OCV state, uniform distributions of V and Te are observed but without S and Zn (Figure S5). The higher increasing intensities for V and Te at the first sputtering time of ~200 s (Figure S5d and S5e) may be attributed to the uneven surface. In contrast, for the MVT-M1 cathode in the discharged state, Figure S5c shows that the sulphur (S) signals originating from the $(CF_3SO_3)^-$ ions in the interlayers of $Zn(OH)_2$ with some $(CF_3SO_3)^-$ ions in the interlayers (abbreviated as $Zn(OH)_2$-S) (evidenced by Figure S10) significantly decrease with the sputtering time (i.e., with increasing depth), directly demonstrating the presence of $Zn(OH)_2$-S on the surface of the cathode rather than the bulk. In addition, the enrichment of Zn and a decrease in the V and Te contents at the surface further prove that the MVT-M1 cathode was covered by a layer of $Zn(OH)_2$-S, which is consistent with the ex situ XPS and SEM results (Figure S6 and S10). Furthermore, the Zn signals in the bulk of the discharged cathode are uniform and of high intensity (Figure S5a and S5b) indicating the insertion of Zn into the MVT-M1 tunnels. After charging, the Zn signals in the bulk of the cathode decrease significantly (Figure S5b), suggesting the extraction of $Zn^{2+}$; the concomitant decrease in S signals indicates a decrease in $Zn(OH)_2$-S and the extraction of $H^+$ (Figure S5c). Because $Zn(OH)_2$-S appears only on the surface of the cathode, interface modification has a particularly promising effect on the morphology and crystallinity of $Zn(OH)_2$-S, thus regulating its reversibility and $H^+$ contribution in ZIBs.[3]

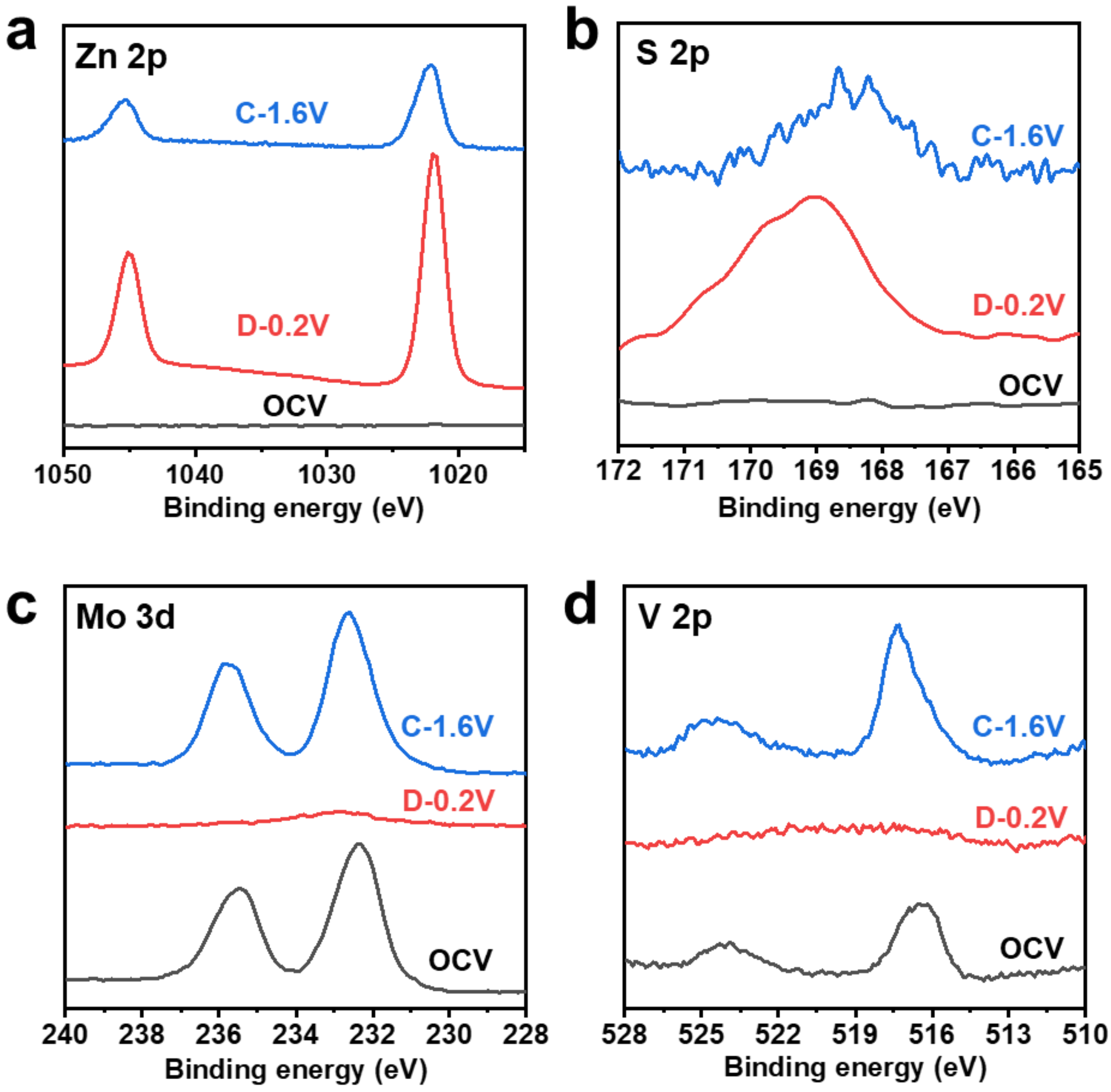


Figure S6. Zn-2p, S-2p, Mo-3d, and V-2p XPS spectra for the MVT-M1 cathode at different states (OCV, D-0.2V, C-1.6V).

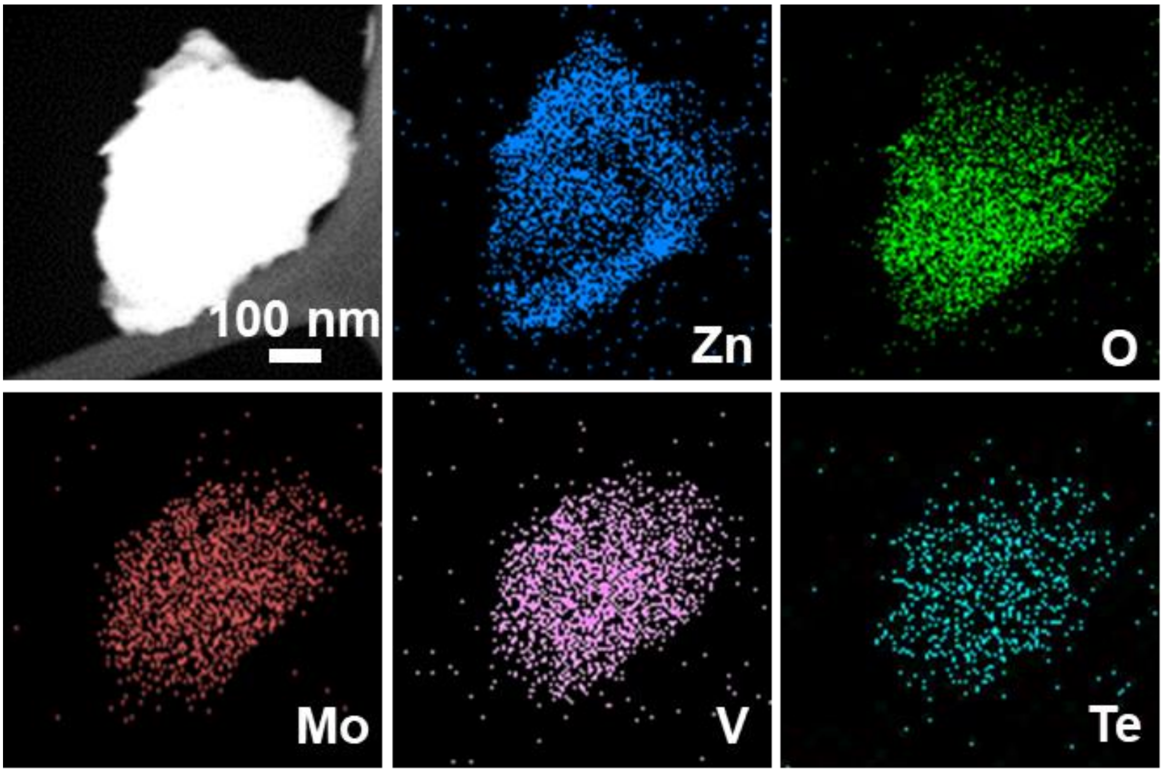


Figure S7. STEM image and corresponding elemental mapping of MVT-M1 in the discharged state (D-0.2V).

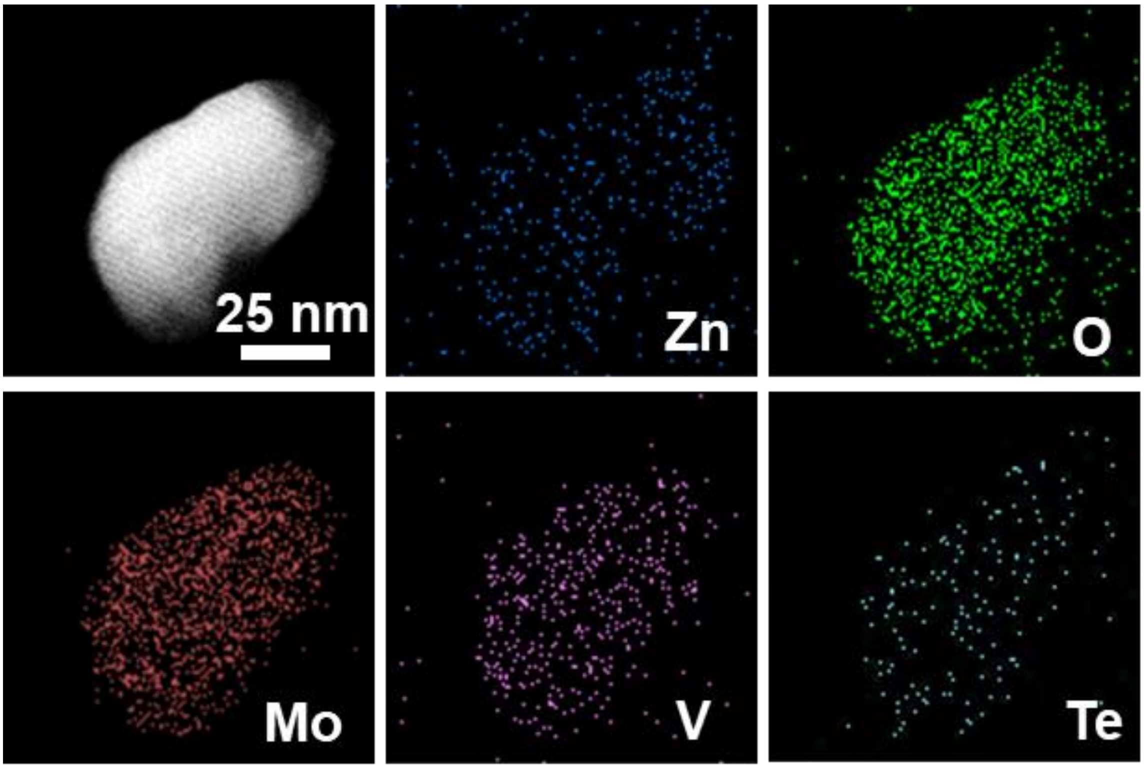


Figure S8. STEM image and corresponding elemental mapping of MVT-M1 in the fully charged state (C-1.6V).

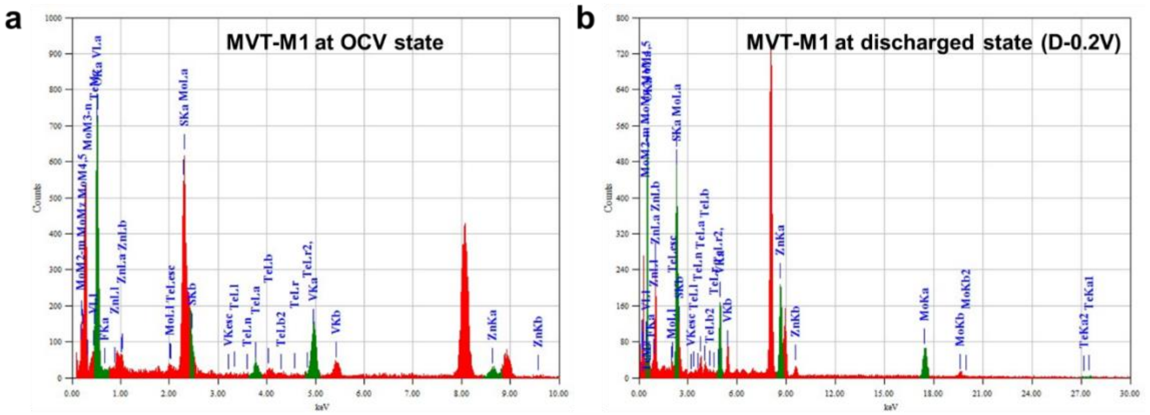


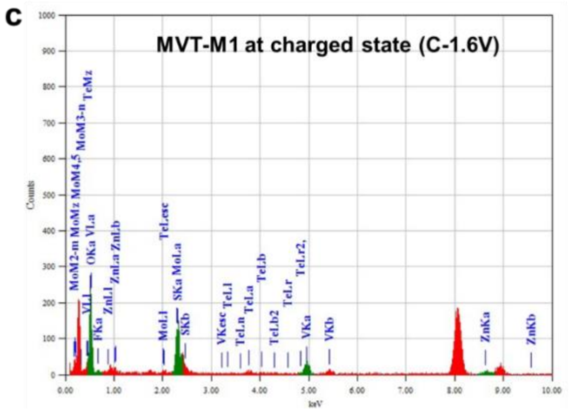


d

| Sample | Zn (atom %) | Mo (atom %) | Zn/Mo atomic ratio |
|---|---|---|---|
| OCV | 2.44 | 16.39 | 0.15:1 |
| D-0.2V | 17.51 | 20.23 | 0.87:1 |
| C-1.6V | 4.84 | 17.39 | 0.28:1 |

Figure S9 EDS spectra for MVT-M1 nanorods in the (a) OCV state, (b) discharged state (D-0.2V) and (c) charged state (C-1.6V). (d) The atomic percent and ratios of Zn and Mo for MVT-M1 nanorods at the OCV state, discharged state (D-0.2V) and charged state (C-1.6V).

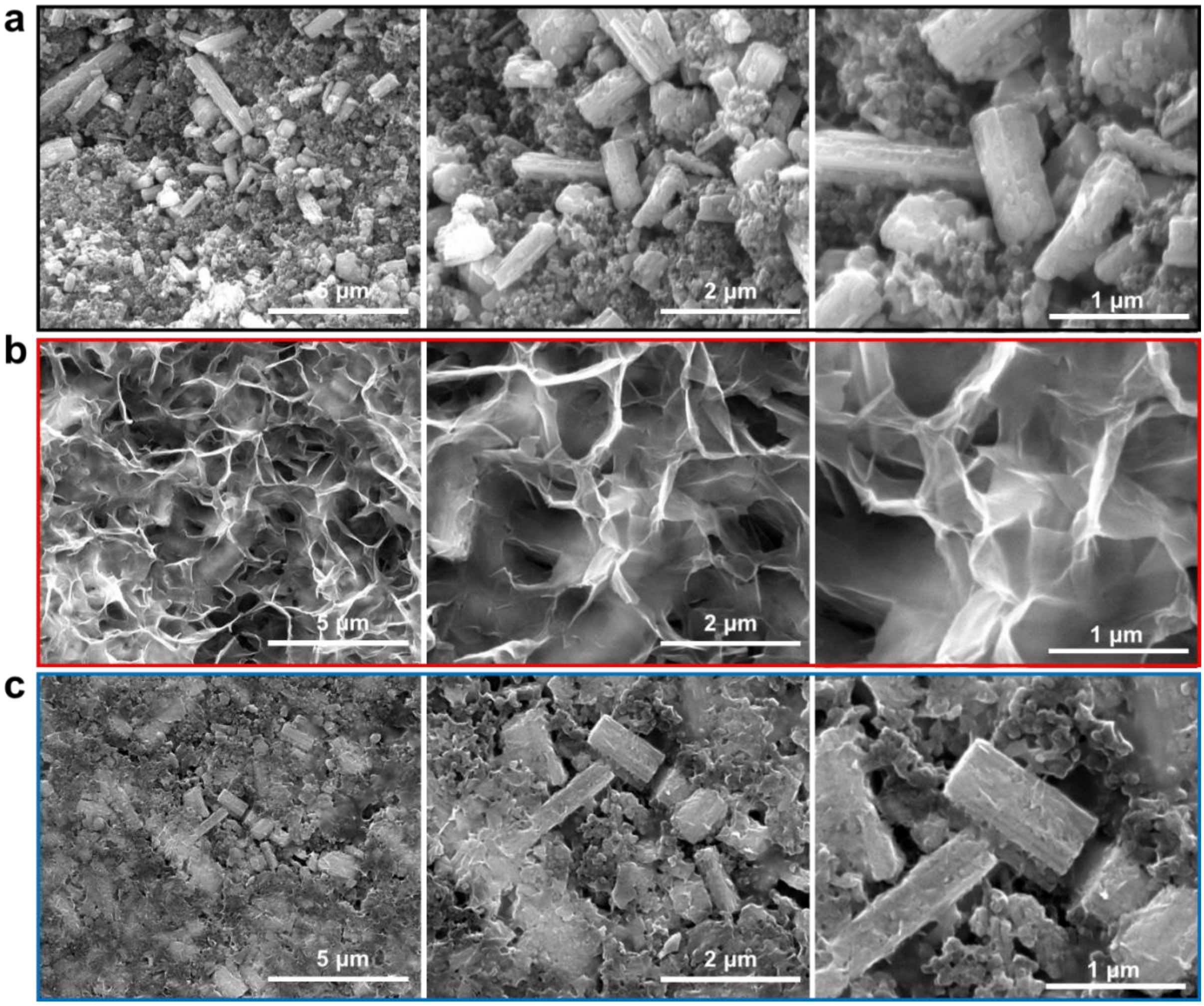


Figure S10. SEM images of the MVT-M1 cathode at various SOCs. (a) OCV state; (b) discharged state (D-0.2V); (c) charged state (C-1.6V).

Compared to MVT-M1 in the OCV state (Figure S10a), many nanoflakes appear on the surface of MVT-M1 in the discharged state (Figure S10b) and then mostly disappear in the charged state (Figure S10c).

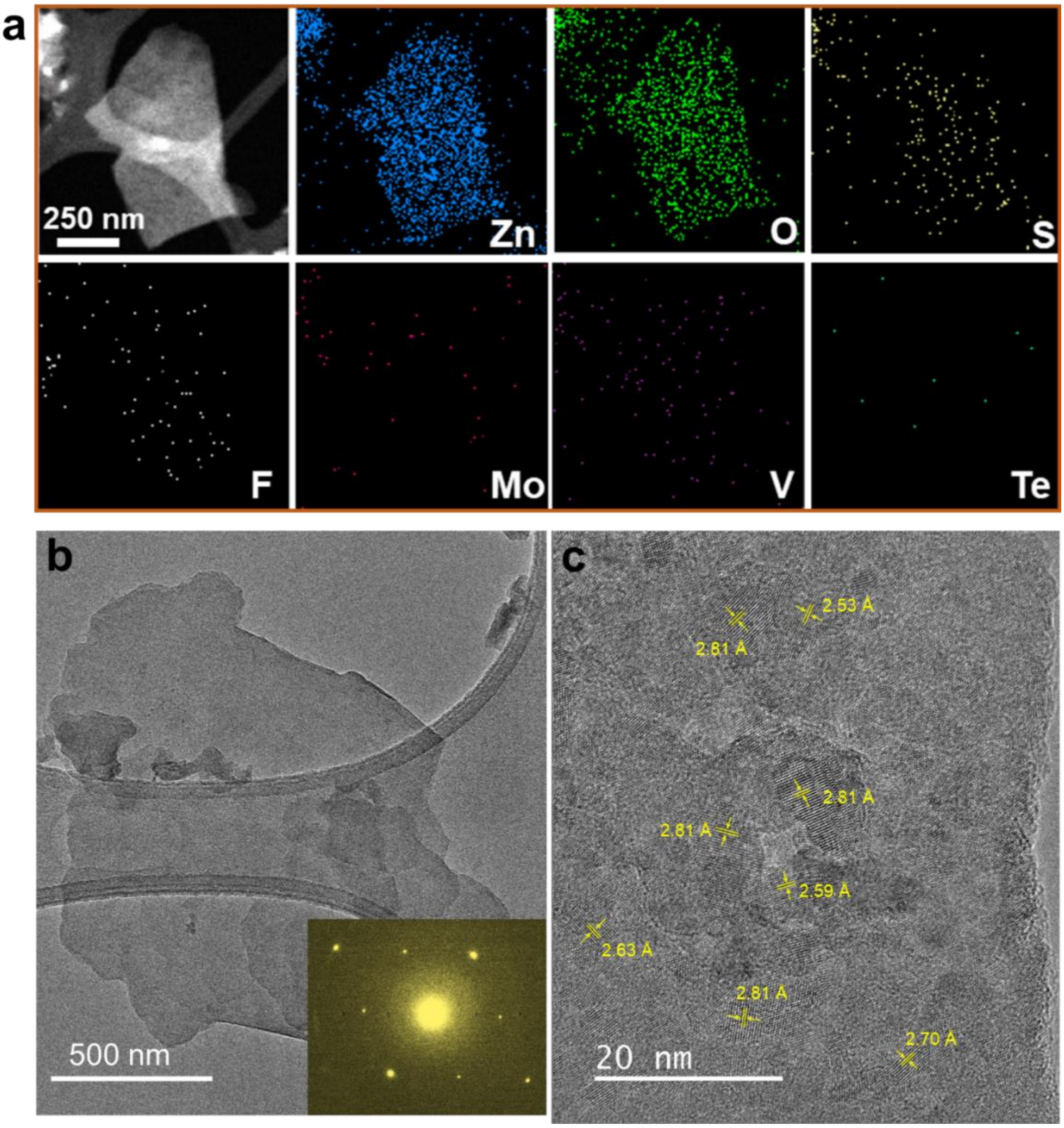


Figure S11. (a) STEM image and corresponding elemental mapping of nanoflakes appearing on the surface of MVT-M1 cathode in the discharged state. (b) TEM of nanoflakes obtained from a fully discharged MVT-M1 cathode (D-0.2V). Inset is the corresponding selected area electron diffraction (SAED). (c) HRTEM image of the nanoflakes on the surface of the MVT-M1 cathode in the discharged state.

The nanoflakes, which were not detected by XRD, are polycrystals comprising small nanocrystals illustrated in the high-resolution (HR) TEM images (Figures S11b and S11c), and they contain mainly Zn and O with minor S and F, as determined from the STEM mappings (Figure S11a). According to previous studies, the nanoflakes were mainly composed of layered $Zn(OH)_2$ with some $(CF_3SO_3)^-$ ions in the interlayers.[4] The presence of $Zn(OH)_2$-S is an indication of $H^+$ insertion into MVT-M1, as the decrease in $H^+$ concentration in the electrolyte

results in an increase in $OH^-$ concentration and ultimately the formation of $Zn(OH)_2$-S at the electrode interface.[4a, 5] Note that a small amount of $Zn(OH)_2$-S remains on the surface of MVT-M1 in the fully charged state, indicating that some inserted $H^+$ ions could not be extracted from the tunnels of MVT-M1.

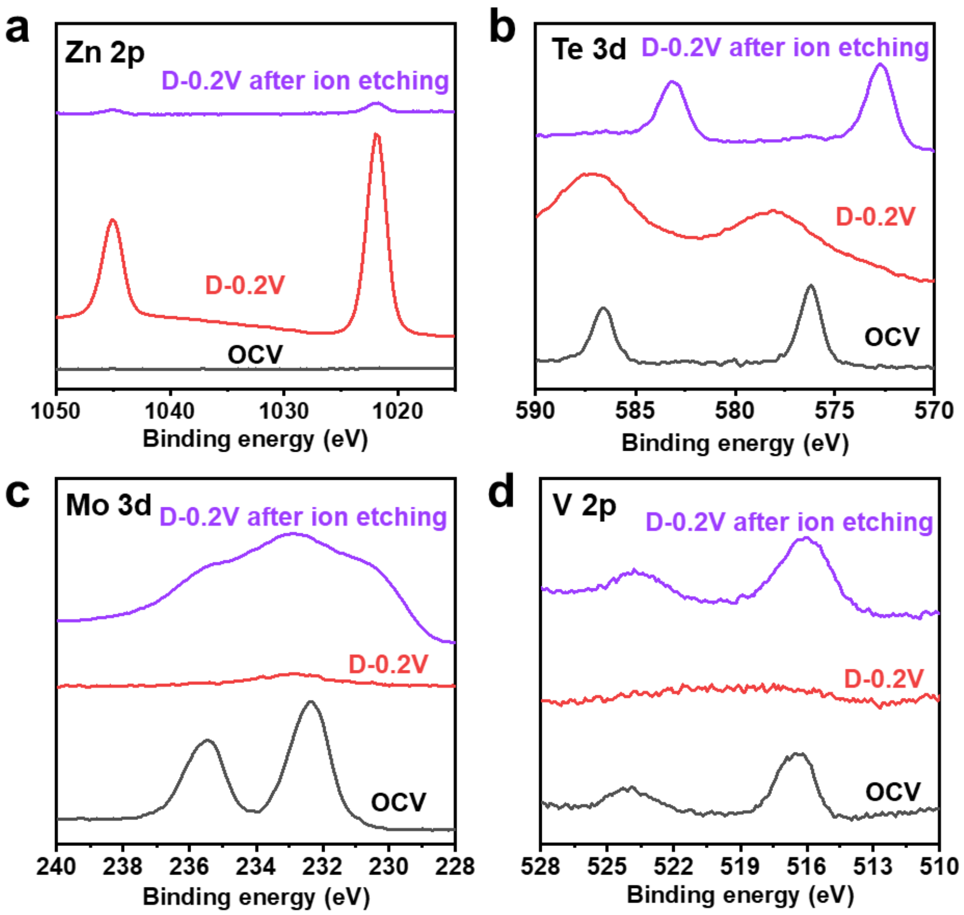


Figure S12. XPS spectra of (a) Zn-2p, (b) Te-3d, (c) Mo-3d and (d) V-2p for MVT-M1 cathodes at the OCV state (OCV), discharged state (D-0.2V), and discharged state after ion etching (D-0.2V after ion etching).

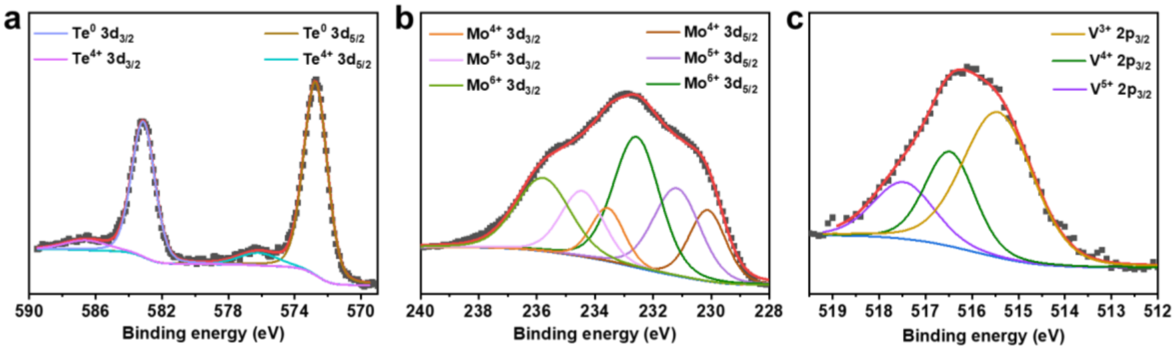


Figure S13. The XPS spectra and valence states of Te, Mo and V elements in MVT-M1 cathodes in the discharged state after ion etching (D-0.2V after ion etching).

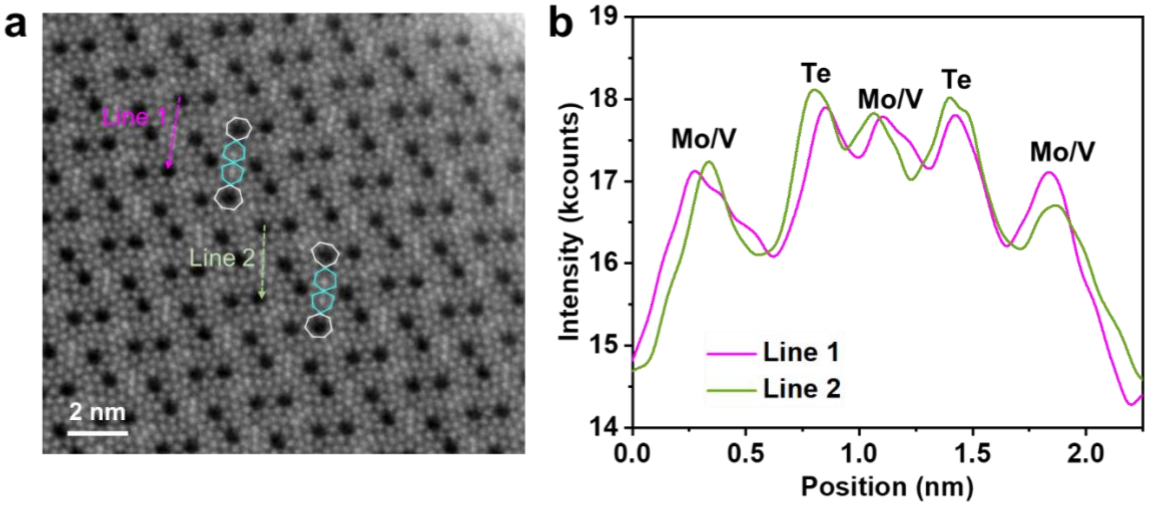


Figure S14. (a) HAADF-STEM image and (b) corresponding atomic intensity along lines 1 and 2 in (a) for MVT-M1 in the OCV state.

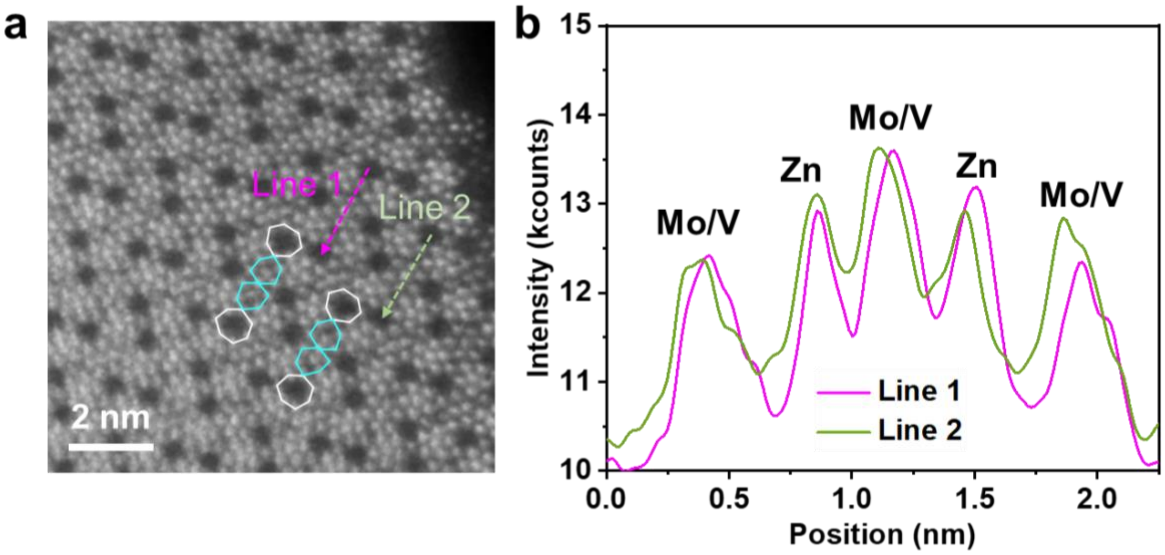


Figure S15. (a) HAADF-STEM image and (b) corresponding atomic intensity along lines 1 and 2 in (a) for MVT-M1 in the discharged state (D-0.2V).

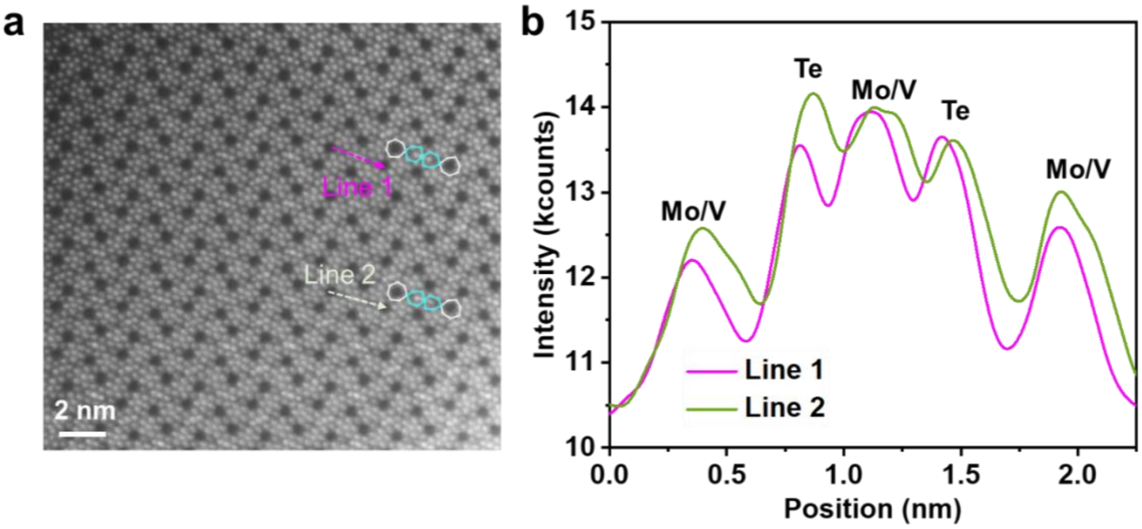


Figure S16. (a) HAADF-STEM image and (b) corresponding atomic intensity along lines 1 and 2 (a) for MVT-M1 in the charged state (C-1.6V).

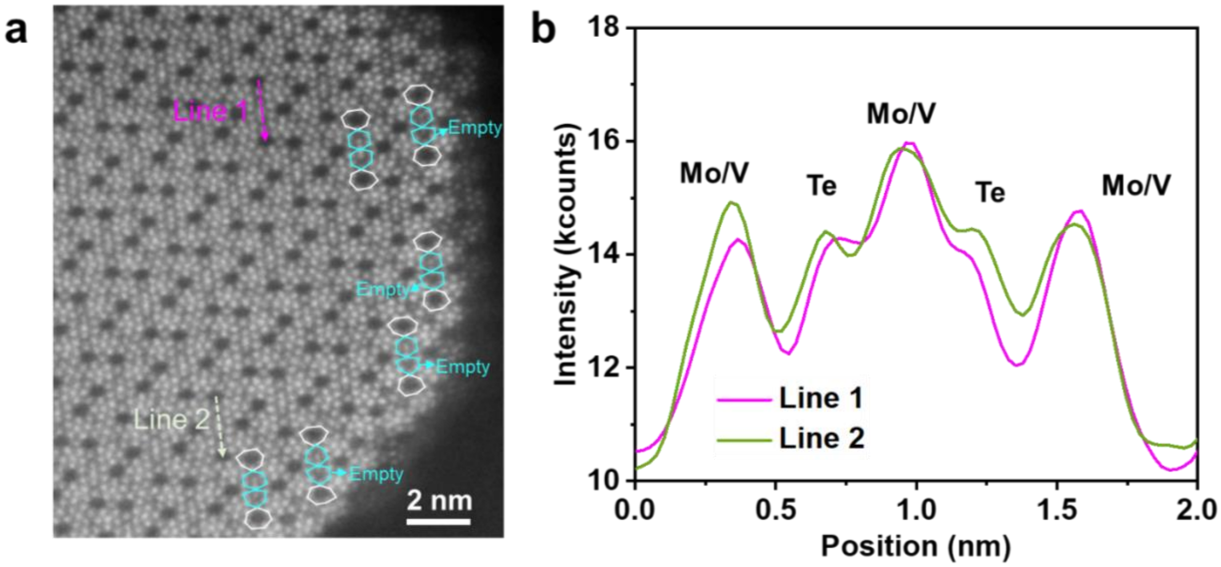


Figure S17. (a) HAADF-STEM image and (b) corresponding atomic intensity along lines 1 and 2 for MVT-M1 after reduction in aqueous $(NH_4)_2SO_4$ (1M) solution.

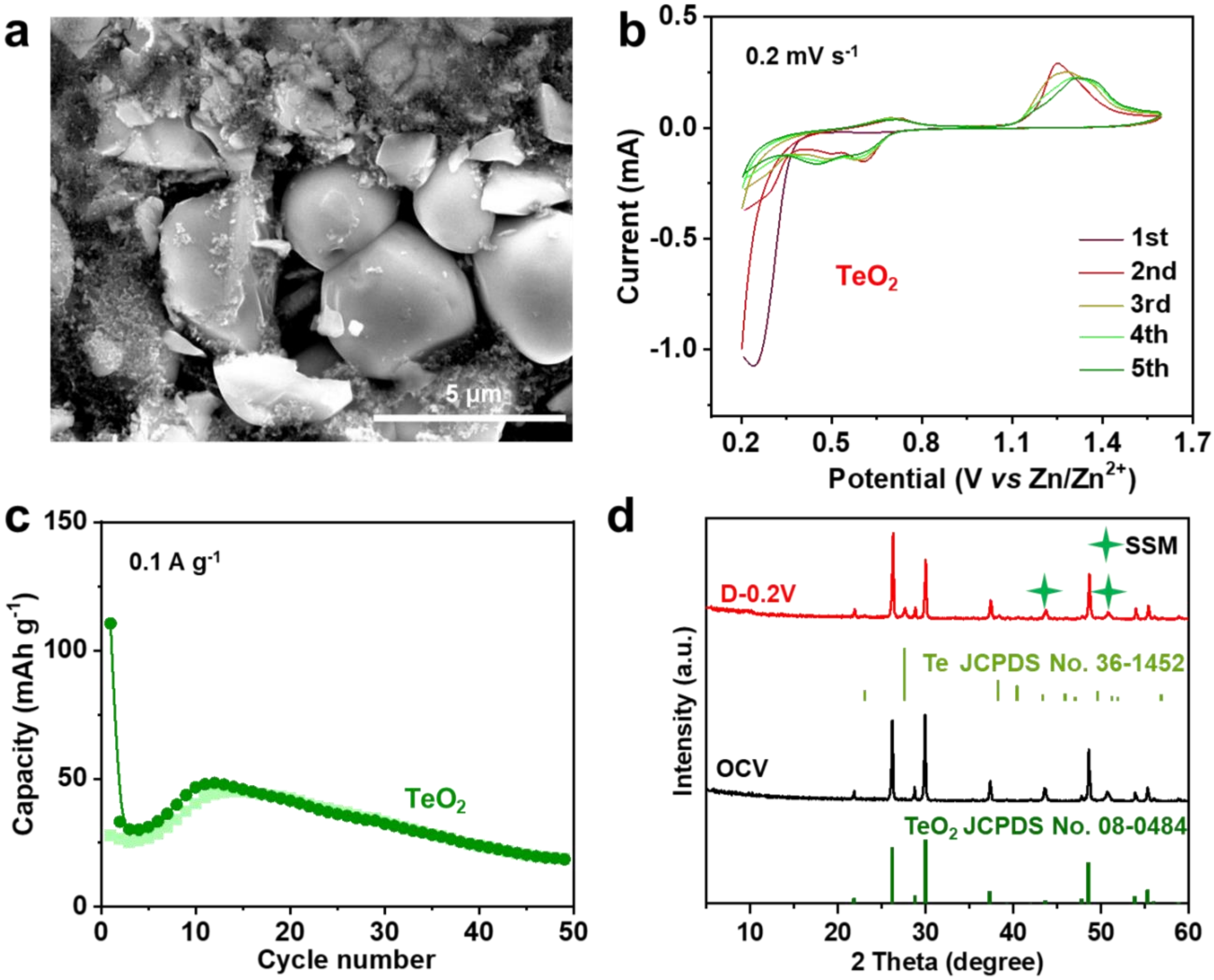


Figure S18. (a) SEM images of the $TeO_2$ cathode. (b) The first five CV curves of the $TeO_2$ cathode in RAZIBs at a scan rate of 0.2 mV $s^{-1}$. (c) The long-term cycling performance at 0.1 A $g^{-1}$ for the $TeO_2$ cathode in RAZIBs. (d) The XRD patterns of the $TeO_2$ cathode at the OCV state and discharged state (D-0.2V).

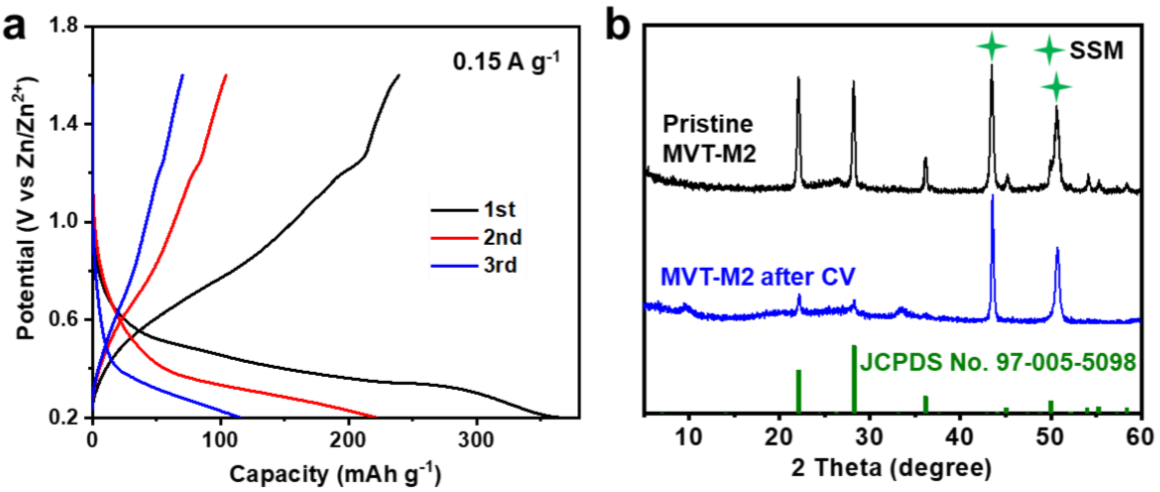


Figure S19. (a) Galvanostatic discharge/charge curves of the first three cycles at 0.15 A $g^{-1}$ for MVT-M2 cathodes in RAZIBs. (b) The XRD patterns of MVT-M2 in the pristine state and after the CV test (5 cycles at 0.2 mV $s^{-1}$).

Note that the higher discharge capacity than charge capacity in the $1^{st}$ cycle indicates the inserted ions cannot be fully extracted when charge.

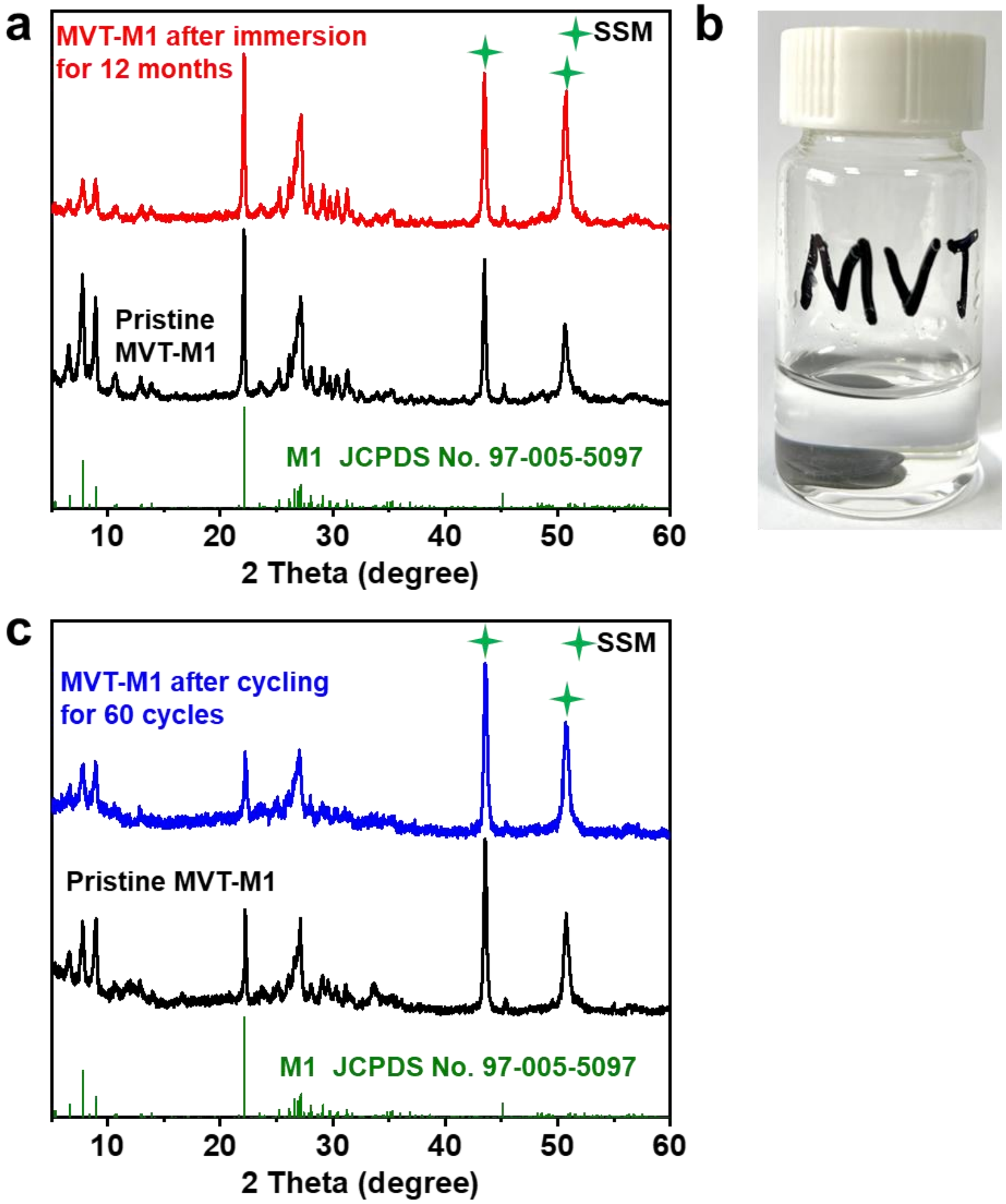


Figure S20. (a) XRD pattern of the MVT-M1 cathode before and after immersion in aqueous $Zn(OTf)_2$ (2M) solution (5 mL) for 12 months. (b) Optical image of the MVT-M1/$Zn(OTf)_2$ system after immersion for 12 months. (c) The XRD patterns of the MVT-M1cathode before and after cycling at 0.15 A $g^{-1}$ for 60 cycles.

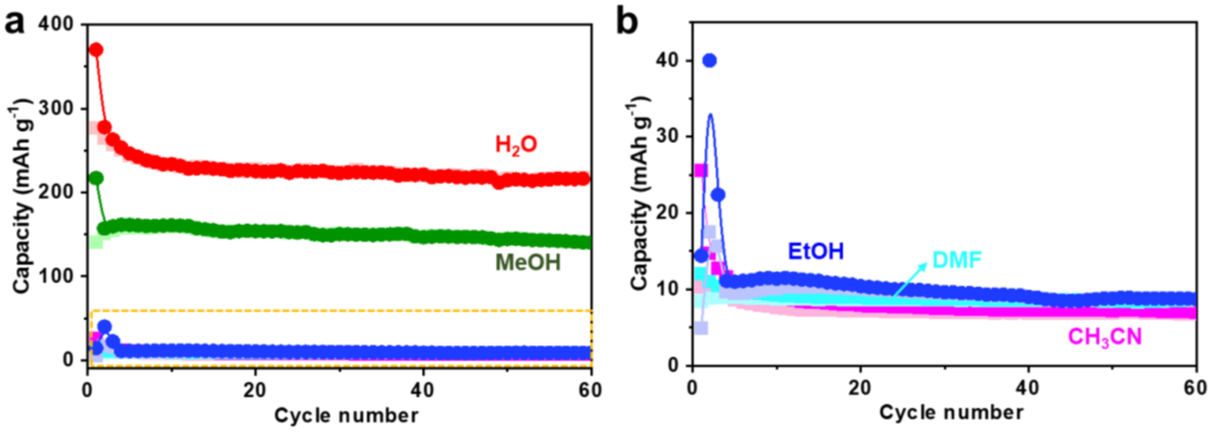


Figure S21. (a) Long-term cycling performance of MVT-M1 in 0.25 M Zn(OTf)$_2$ electrolytes with different solvents at 0.15 A g$^{-1}$. (b) Enlargement of the orange rectangle in Figure S20a.

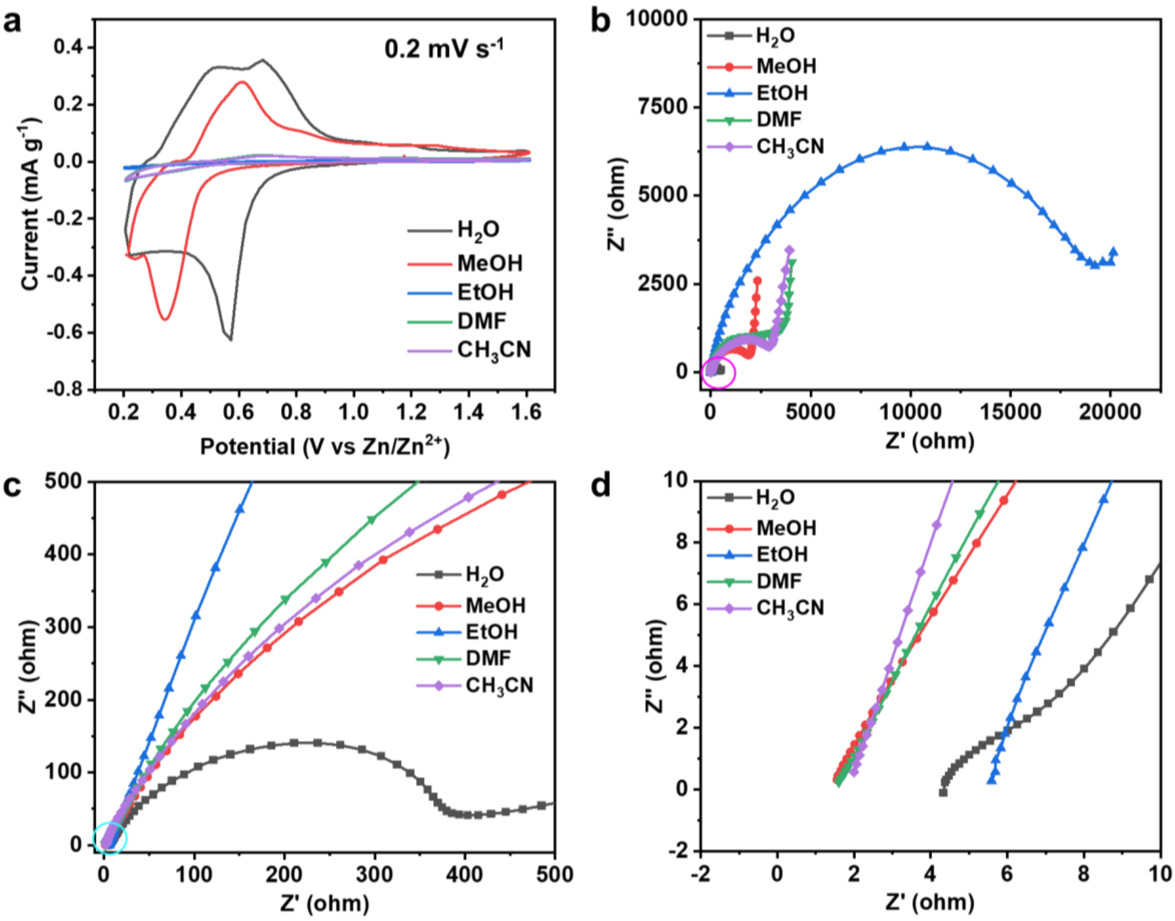


Figure S22. (a) The CV curves of MVT-M1 in the electrolytes with various solvents. (b) EIS spectra at OCV states of Zn||MVT-M1 cells in the electrolytes with various solvents. (c) Enlargement of the magenta circle in Figure b. (d) Enlargement of cyan circle in Figure c.

The ohmic resistances of the battery in the electrolytes using different solvents range from 1.9 to 6 ohm, and the current is about 0.3 mA. Therefore, the IR drop is lower than 2 mV, that is the ionic conductivities in the five electrolytes are sufficiently high.

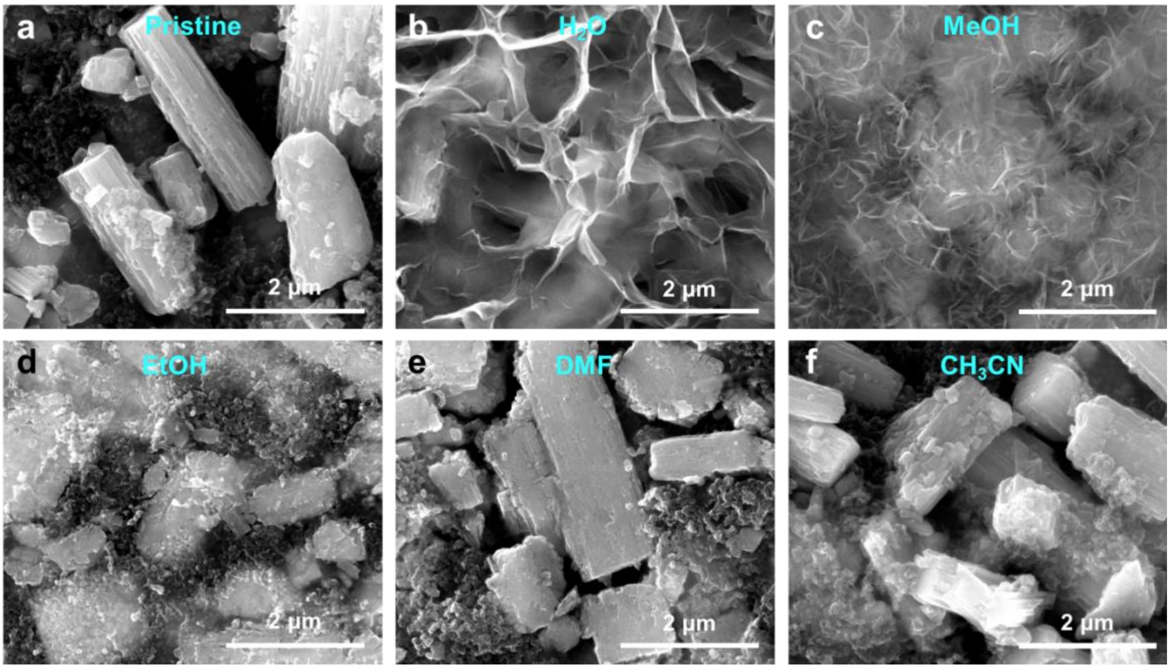


Figure S23. SEM images of MVT-M1 in the (a) pristine state and (b-f) discharged state in the electrolytes using various solvents.

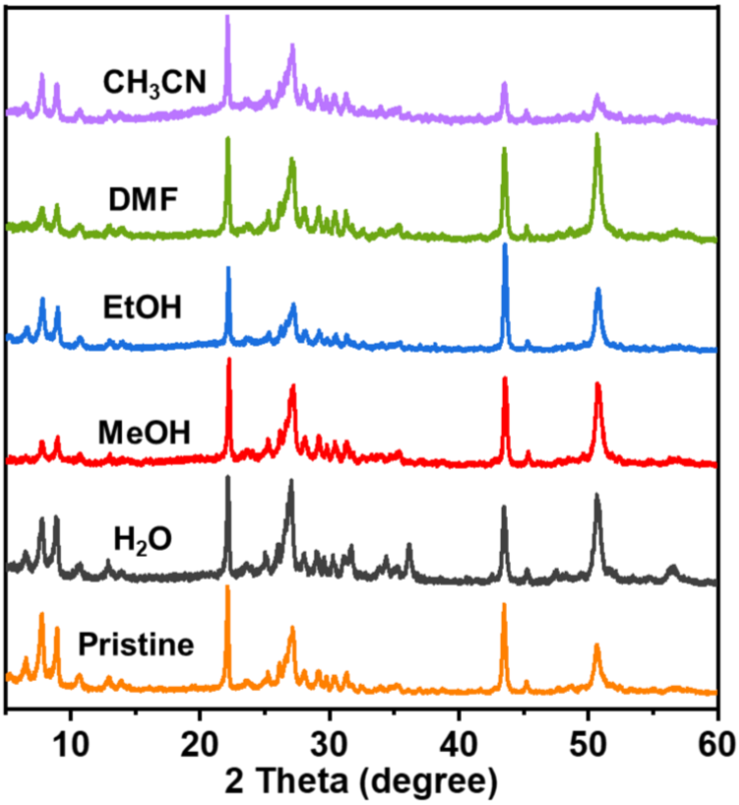


Figure S24. XRD patterns of MVT-M1 in the pristine state and discharged state in electrolytes using various solvents.

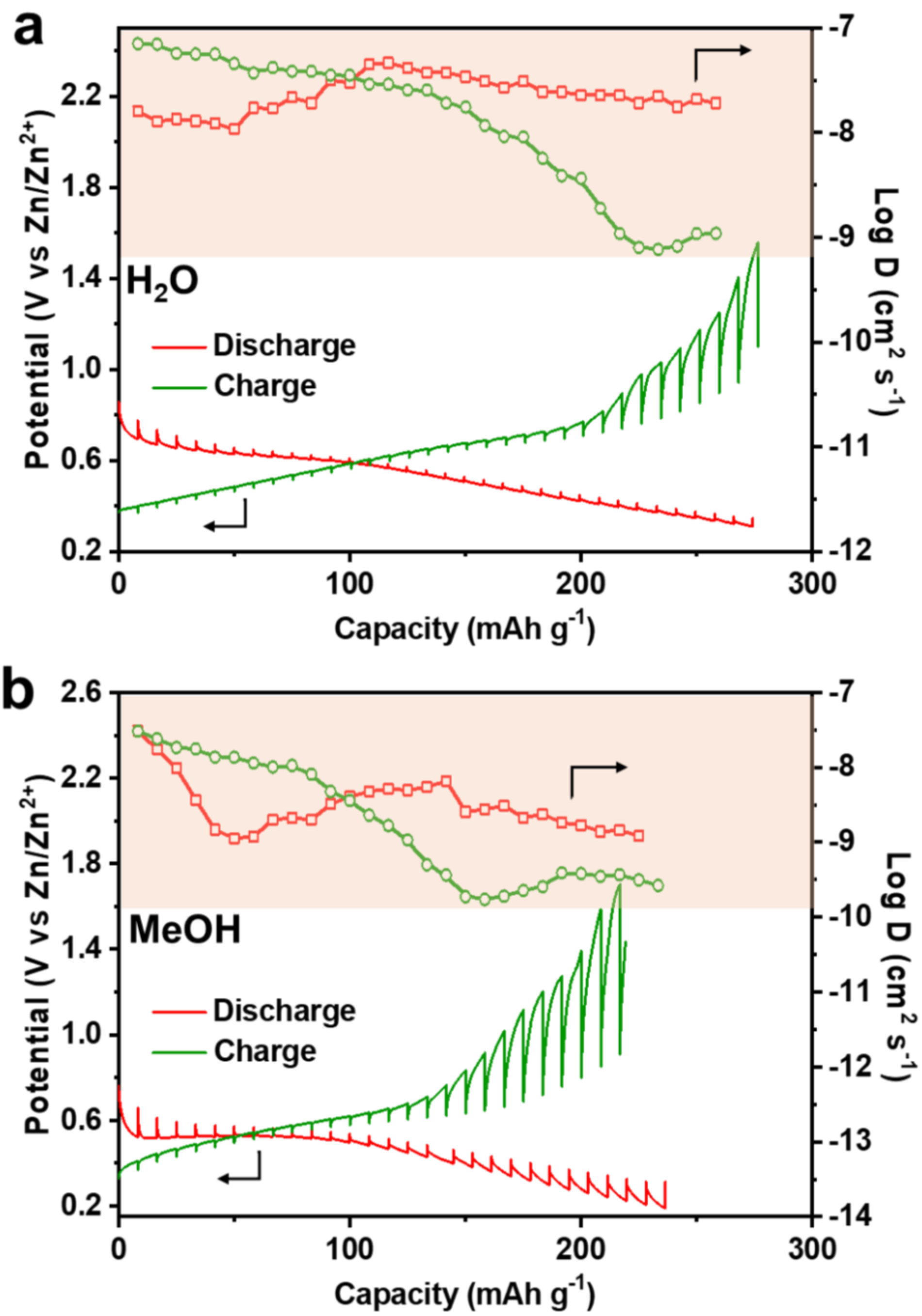


Figure S25. GITT curves and $Zn^{2+}$ diffusion coefficient of the MVT-M1 cathode at a current density of 50 mA $g^{-1}$ in 1 M $Zn(OTf)_2$ electrolyte with solvents of (a) water and (b) methanol.

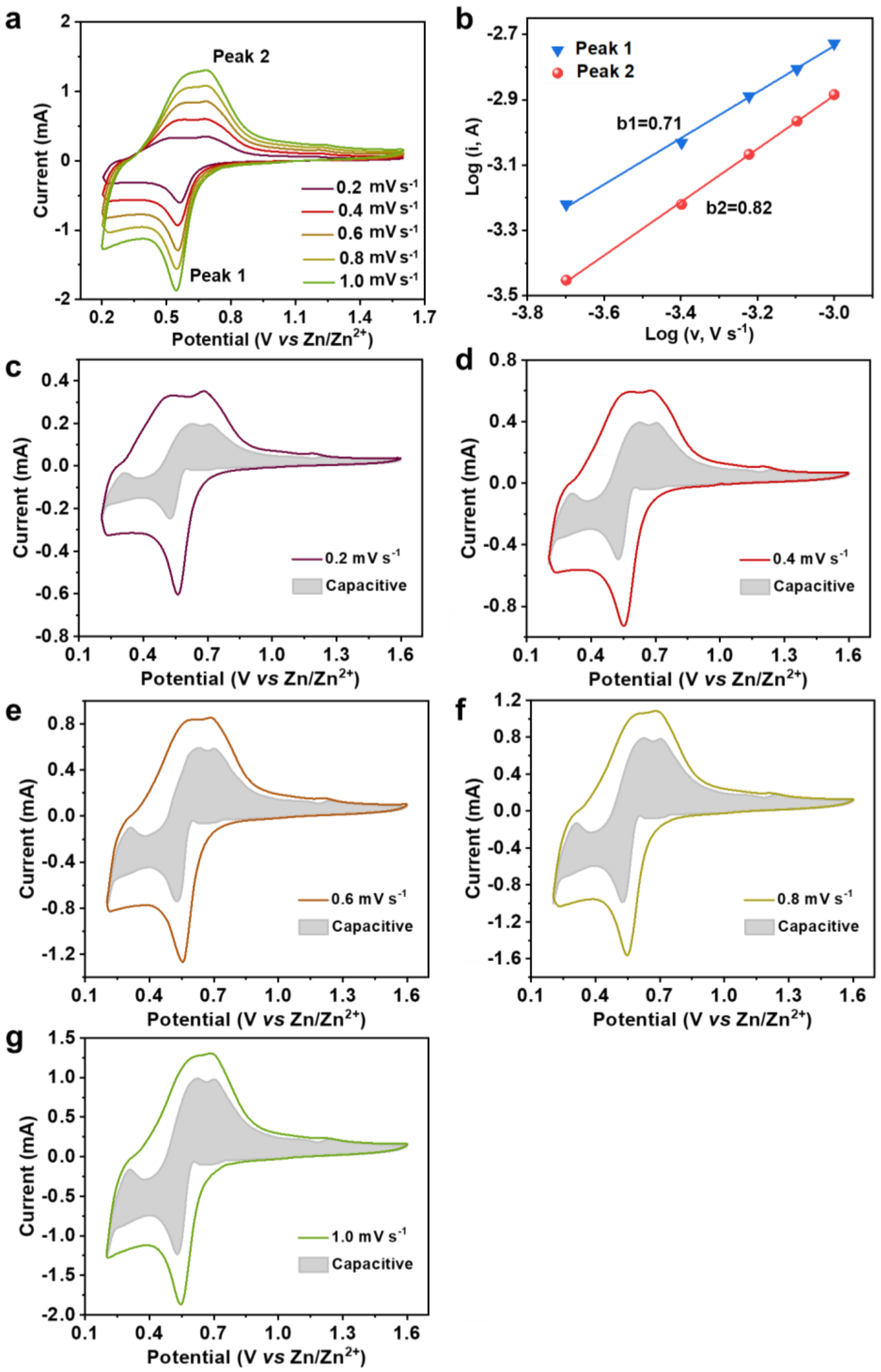


Figure S26. (a) The CV curves of MVT-M1 at multiple scan rates from 0.2 to 1 mV $s^{-1}$. (b) The corresponding plots of log (peak current, i) vs log (scan rate, v) at cathodic and anodic peaks. (c-g) Capacitive contribution (grey portion) and diffusion contribution (white portion) of MVT-M1 at multiple scan rates from 0.2 to 1 mV $s^{-1}$.

Generally, the peak current (i) of a CV can be related to the scan rate (v) using an empirical power-law relationship to describe the combination of surface-controlled capacitive effects ($i_1 = k_1v$) and diffusion-controlled processes ($i_2 = k_2v^{1/2}$) [6],

$$i = k_1 v + k_2 v^{1/2} \approx a v^b, \quad (2)$$

where $k_1$, $k_2$, a, and b are variable parameters with b = 0.5 for a diffusion-controlled charge-transfer process and 1.0 for a surface-controlled capacitive process.

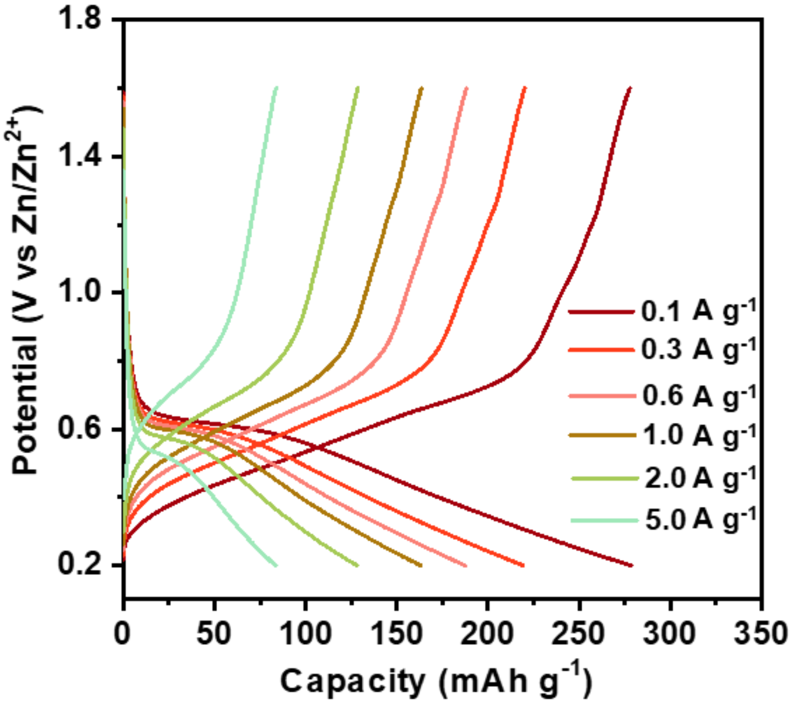


Figure S27. Galvanostatic discharge-charge profiles at different current densities for the MVT-M1 cathode in ZIBs.

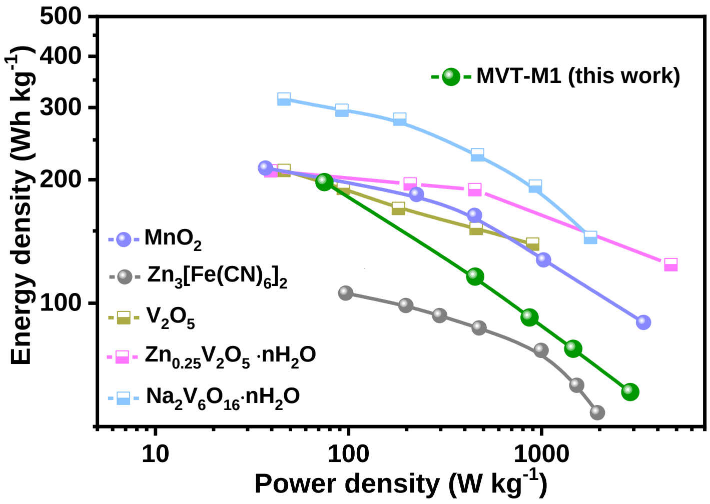


Figure S28 Ragone plot (energy density vs power density) comparing MVT-M1 with tunnel-structured materials ($MnO_2$, $Zn_3[Fe(CN)_6]_2$) and layer-structured materials ($V_2O_5$, $Zn_{0.25}V_2O_5{\cdot}nH_2O$, and $Na_2V_6O_{16}{\cdot}nH_2O$).

As $VO_2$ and $V_2O_3$ with tunnel structures would be oxidized to layered $Zn_xV_2O_5{\cdot}nH_2O$, the Ragone plot of $Zn_xV_2O_5{\cdot}nH_2O$ is provided to represent those for $VO_2$ and $V_2O_3$ with tunnel structures.

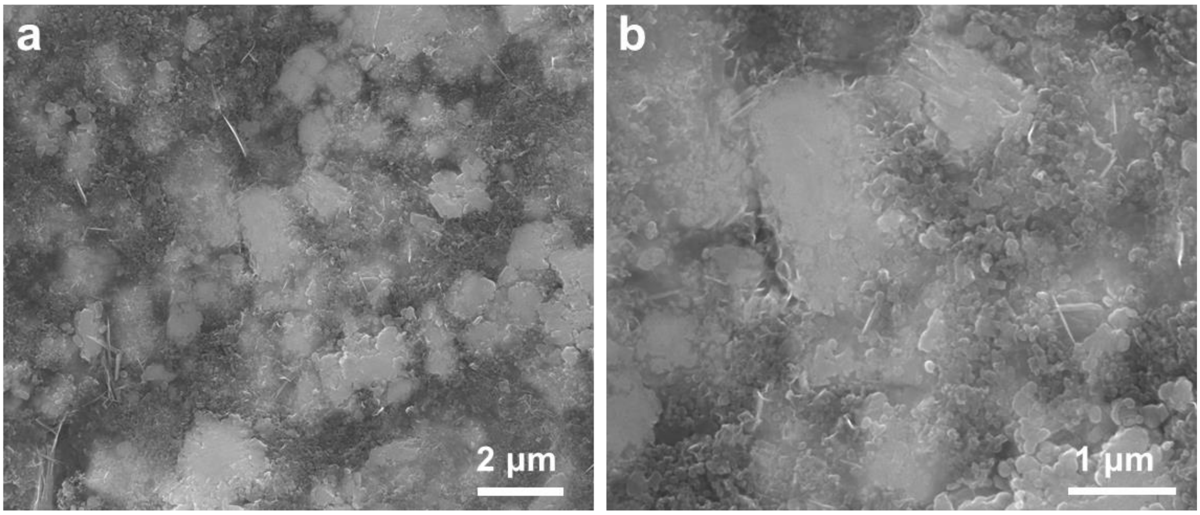


Figure S29 SEM images of MVT-M1 cathodes after 5000 cycles at 5 A $g^{-1}$.

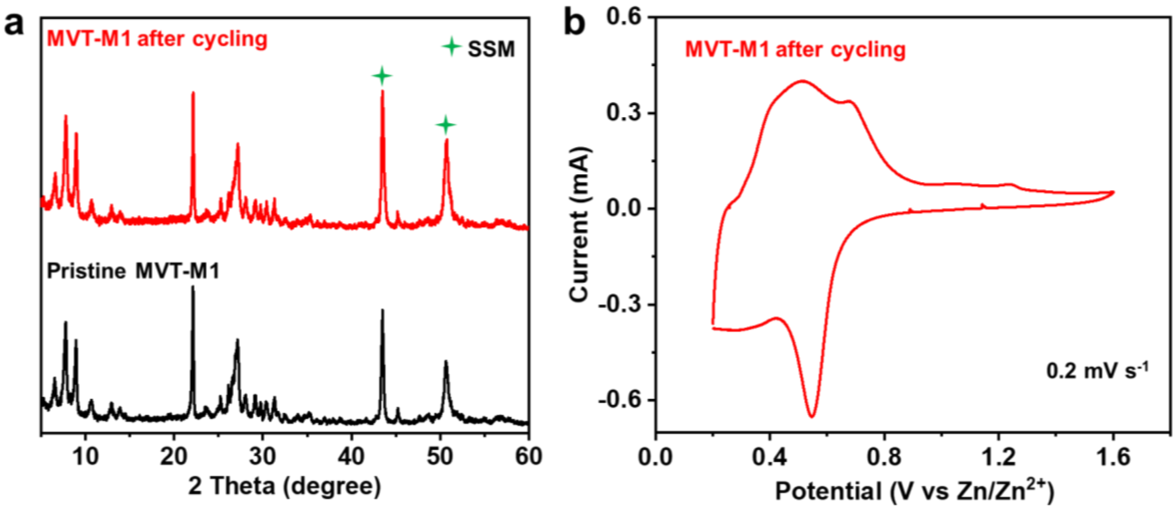


Figure S30. (a) XRD patterns of MVT-M1 in the pristine state and after cycling for 5000 cycles at 5 A $g^{-1}$. (b) CV curve of MVT-M1 after cycling for 5000 cycles at 5 A $g^{-1}$ in ZIBs.

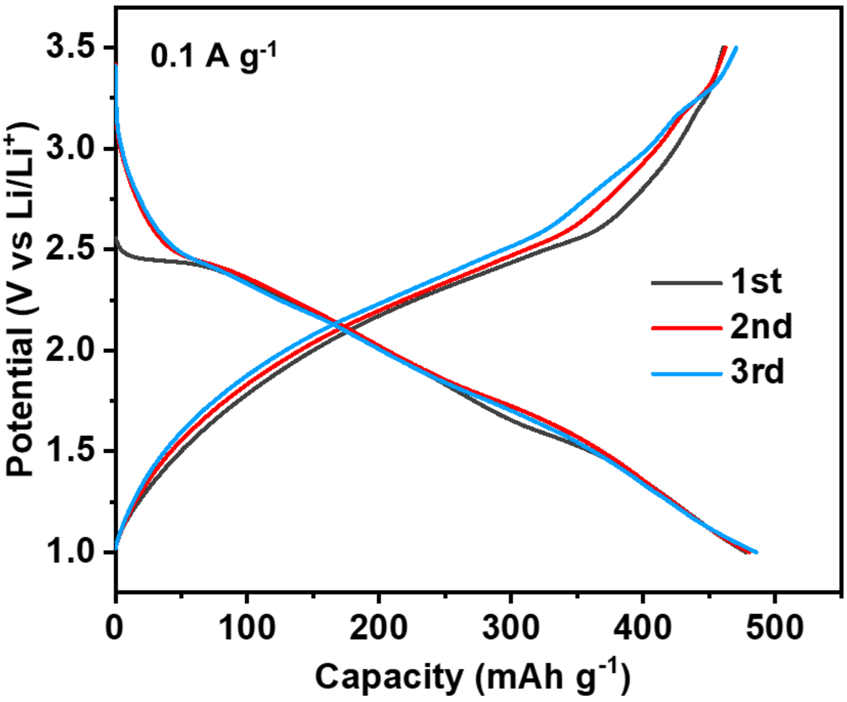


Figure S31. The discharge/charge profiles of the first three cycles at a current density of 0.1 A $g^{-1}$ for MVT-M1 in Li metal batteries (electrolyte: 1.0 M $LiPF_6$ in ethylene carbonate and diethyl carbonate).

Table S1 The XRF results of MVT-M1.

| mass | Concentration (%) |
|---|---|
| Mo | 45.55 |
| V | 9.896 |
| Te | 7.068 |

Note: the MVT-M1 mass for XRF measurement was 4.015 g.

Table S2 Ionic conductivities and electrolyte resistances of 0.25 M $Zn(OTf)_2$ electrolytes in coin cells (CR2032) using various solvents including $H_2O$, MeOH, EtOH, DMF, and $CH_3CN$.

| Solvents | Ionic conductivity ($mS\ cm^{-1}$) | Electrolyte resistance (ohm) |
|---|---|---|
| $H_2O$ | 29.1 | 0.22 |
| MeOH | 14.56 | 0.45 |
| EtOH | 2.79 | 0.44 |
| DMF | 14.90 | 2.32 |
| $CH_3CN$ | 15.92 | 0.41 |

Note: the electrolyte resistances were determined based on a distance of 100 μm between anode and cathode, and an area of 1.54 $cm^2$.

Table S3. Comparison of the electrochemical performance for the MVT-M1 and some representative cathode materials in ZIBs.

| Cathode material | Electrolyte | Specific capacity | Cycle performance | Ref. |
|---|---|---|---|---|
| **MVT-M1** | 2 M $Zn(CF_3SO_3)_2$ | 300 mAh g-1 at 0.1 A g-1 | 96 mAh $g^{-1}$ at 5 A $g^{-1}$<br>88% retained after 5,000 cycles at 5 A $g^{-1}$ | This work |
| **α-$MnO_2$** | 1 M $ZnSO_4$ | 210 mAh $g^{-1}$at 0.5 C | 133 mAh $g^{-1}$at 6 C (~1.2 A $g^{-1}$)<br>77% retained after 100 cycles at 6 C | [S7] |
| **β-$MnO_2$** | 3 M $Zn(CF_3SO_3)_2$ + 0.1 $Mn(CF_3SO_3)_2$ | 258 mAh $g^{-1}$at 0.2 A $g^{-1}$ | 144 mAh $g^{-1}$at 2 $g^{-1}$<br>94% retained after 2,000 cycles at 2 A $g^{-1}$ | [S8] |
| **$ZnMn_2O_4$+C** | 3 M $Zn(CF_3SO_3)_2$ | 150mAh $g^{-1}$at 0.05 A $g^{-1}$ | 72 mAh $g^{-1}$ at 2 A $g^{-1}$<br>94% retained after 500 cycles at 0.5 A $g^{-1}$ | [S9] |
| **$Zn_3[Fe(CN)_6]_2$** | 1 M $ZnSO_4$ | 65.4 mAh $g^{-1}$at 0.06 A $g^{-1}$ | 53 mAh $g^{-1}$at 0.3 A $g^{-1}$<br>81% retained after 100 cycles at 0.3A $g^{-1}$ | [S10] |
| **$Mo_6S_8$** | 0.1 M $ZnSO_4$ | 88 mAh $g^{-1}$at 0.0064 A $g^{-1}$ | 57 mAh $g^{-1}$at 0.128 A $g^{-1}$<br>no cycling performance | [S11] |
| **Quinone** | 3 M $Zn(CF_3SO_3)_2$ | 335 mAh $g^{-1}$at 0.02 A $g^{-1}$ | ~150 mAh $g^{-1}$ at 0.5 A $g^{-1}$<br>87% retained after 1,000 cycles at 0.5 A $g^{-1}$ | [S12] |
| **α-$V_2O_5$** | 21 M bis(trifluoromethanesulfonyl)imide + 1 M $Zn(CF_3SO_3)_2$ | 242 mAh $g^{-1}$at 0.05 A $g^{-1}$ | 156 mAh $g^{-1}$at 1 A $g^{-1}$<br>80% retained after 2,000 cycles at 1 A $g^{-1}$ | [S13] |
| **$V_2O_5 \cdot nH_2O$ @graphene** | 3 M $Zn(CF_3SO_3)_2$ | 372 mAh$g^{-1}$ at 0.3 A $g^{-1}$ | 290 mAh $g^{-1}$at 6 A $g^{-1}$<br>71% retained after 500 cycles at 6 A $g^{-1}$ | [S14] |
| **$Zn_{0.25}V_2O_5 \cdot nH_2O$** | 1 M $ZnSO_4$ | 282 mAh$g^{-1}$ at 0.3 A $g^{-1}$ | 260 mAh $g^{-1}$at 2.4 A $g^{-1}$<br>80% retained after 1,000 cycles at 2.4 A $g^{-1}$ | [S15] |
| **$H_2V_3O_8$@ Graphene** | 3 M $Zn(CF_3SO_3)_2$ | 394 mAh $g^{-1}$at 0.1 A $g^{-1}$ | 270 mAh $g^{-1}$at 6 A $g^{-1}$<br>87% retained after 1,000 cycles at 6 A $g^{-1}$ | [S16] |
| **$VS_2$** | 1 M $ZnSO_4$ | 190 mAh $g^{-1}$at 0.05 A $g^{-1}$ | 138 mAh 52$g^{-1}$ at 0.5 $g^{-1}$<br>80% retained after 200 cycles at 0.5 A $g^{-1}$ | [S17] |

## References


1 (a) A. de Arriba, B. Solsona, E. García-González, P. Concepción, J. M. L. Nieto, *Appl. Catal. A-Gen.*, 2022, **643**, 118780; (b) J. Holmberg, R. K. Grasselli, A. Andersson, *Appl. Catal. A-Gen.*, 2004, **270**, 121-134.

2 X. Guo, G. Fang, W. Zhang, J. Zhou, L. Shan, L. Wang, C. Wang, T. Lin, Y. Tang, S. Liang, *Adv. Energy Mater.*, 2018, **8**, 1801819.

3 J. Guo, J. Ming, Y. Lei, W. Zhang, C. Xia, Y. Cui, H. N. Alshareef, *ACS Energy Lett.*, 2019, **4**, 2776-2781.

4 (a) K. Zhu, T. Wu, S. Sun, W. van den Bergh, M. Stefik, K. Huang, *Energy Storage Mater.*, 2020, **29**, 60-70. (b) A. Gordeeva, Y. J. Hsu, I. Z. Jenei, P. H. B. Brant Carvalho, S. I. Simak, O. Andersson, U. Haussermann, *ACS Omega*, 2020, **5**, 17617-17627.

5 J. Huang, Z. Wang, M. Hou, X. Dong, Y. Liu, Y. Wang, Y. Xia, *Nat. Commun.*, 2018, **9**, 2906.

6 V. Augustyn, P. Simon, B. Dunn, *Energy Environ. Sci.*, **2014**, *7*, 1597. (b) C. Choi, D. S. Ashby, D. M. Butts, R. H. DeBlock, Q. Wei, J. Lau, B. Dunn, *Nat. Rev. Mater.*, **2019**, *5*, 5.

7 C. Xu, B. Li, H. Du, F. Kang, *Angew. Chem. Int. Ed.*, **2012**, *51*, 933.

8 N. Zhang, F. Cheng, J. Liu, L. Wang, X. Long, X. Liu, F. Li, J. Chen, *Nat. Commun.*, **2017**, *8*, 405.

9 N. Zhang, F. Cheng, Y. Liu, Q. Zhao, K. Lei, C. Chen, X. Liu, J. Chen, *J. Am. Chem. Soc.*, **2016**, *138*, 12894.

10 L. Zhang, L. Chen, X. Zhou, Z. Liu, *Adv. Energy Mater.*, 2015, **5**, 1400930.

11 C. S. Munseok, H. W. Jongwook, L. Sung-Chul, H. Seung-Tae, *Inorg. Chem.*, **2016**, *55*, 3294.

12 Q. Zhao, W. Huang, Z. Luo, L. Liu, Y. Lu, Y. Li, L. Li, J. Hu, H. Ma, J. Chen, *Sci. Adv.*, **2018**, *4*, eaao1761.

13 P. Hu, M. Yan, T. Zhu, X. Wang, X. Wei, J. Li, L. Zhou, Z. Li, L. Chen, L. Mai, *ACS Appl. Mater. Interfaces*, **2017**, *9*, 42717.

14 M. Yan, P. He, Y. Chen, S. Wang, Q. Wei, K. Zhao, X. Xu, Q. An, Y. Shuang, Y. Shao, K. T. Mueller, L. Mai, J. Liu, J. Yang, *Adv. Mater.*, **2018**, *30*, 1703725.

15 D. Kundu, B. D. Adams, V. Duffort, S. H. Vajargah, L. F. Nazar, *Nat. Energy*, **2016**, *1*, 16119.

16 Q. Pang, C. Sun, Y. Yu, K. Zhao, Z. Zhang, P. M. Voyles, G. Chen, Y. Wei, X. Wang, *Adv. Energy Mater.*, **2018**, *8*, 1800144.

17 P. He, M. Yan, G. Zhang, R. Sun, L. Chen, Q. An, L. Mai, *Adv. Energy Mater.*, **2017**, *7*, 1601920.
